# Conduction through a narrow inward-rectifier K^+^ channel pore

**DOI:** 10.1085/jgp.201912359

**Published:** 2019-09-11

**Authors:** Harald Bernsteiner, Eva-Maria Zangerl-Plessl, Xingyu Chen, Anna Stary-Weinzinger

**Affiliations:** Department of Pharmacology and Toxicology, University of Vienna, Vienna, Austria

## Abstract

Inwardly rectifying potassium (Kir) channels play a key role in controlling membrane potentials in excitable and unexcitable cells, thereby regulating a plethora of physiological processes. G-protein–gated Kir channels control heart rate and neuronal excitability via small hyperpolarizing outward K^+^ currents near the resting membrane potential. Despite recent breakthroughs in x-ray crystallography and cryo-EM, the gating and conduction mechanisms of these channels are poorly understood. MD simulations have provided unprecedented details concerning the gating and conduction mechanisms of voltage-gated K^+^ and Na^+^ channels. Here, we use multi-microsecond–timescale MD simulations based on the crystal structures of GIRK2 (Kir3.2) bound to phosphatidylinositol-4,5-bisphosphate to provide detailed insights into the channel’s gating dynamics, including insights into the behavior of the G-loop gate. The simulations also elucidate the elementary steps that underlie the movement of K^+^ ions through an inward-rectifier K^+^ channel under an applied electric field. Our simulations suggest that K^+^ permeation might occur via direct knock-on, similar to the mechanism recently shown for K_v_ channels.

## Abstract (precis)

G-protein–gated inwardly rectifying potassium channels are important mediators of inhibitory neurotransmission. Based on microsecond-scale molecular dynamics simulations, Bernsteiner et al. propose novel gating details that may enable K^+^ flux via a direct knock-on mechanism.

## Introduction

Inwardly rectifying potassium (Kir) channels regulate potassium flux through otherwise ion-impermeable cell membranes. Under physiological conditions, these channels enable large K^+^ influx at potentials negative to the equilibrium potential of potassium but permit little outward current at potentials positive to the equilibrium potential of potassium, due to block of outward K^+^ flux by Mg^2+^ and polyamines. This results in a small hyperpolarizing current at the resting membrane potential (Hibino et al., 2010; Lüscher and Slesinger, 2010). K^+^ conductance is further controlled via regulatory ligands that modulate gating of Kir channels. Phosphatidylinositol-4,5-bisphosphate (PIP_2_) binding to a canonical PIP_2_-binding site is essential for activation of all Kir channels (Xie et al., 2007; Hibino et al., 2010), while additional regulation is distinct for each subfamily. Kir1, Kir4, and Kir4/5 are controlled by pH, Kir3 by Gβγ released from G-protein–coupled receptors and Na^+^ ions, and Kir6 channels by ADP/ATP and sulphonylurea receptors (Ashcroft, 1988; Nichols and Lederer, 1991; Ashcroft and Gribble, 1998; Huang et al., 1998; Aryal et al., 2009; Hibino et al., 2010). Further, bulk anionic lipids have been identified as positive allosteric regulators for Kir2 channels (Cheng et al., 2011; Lee et al., 2013, 2016). Moreover, phosphorylation by protein kinases influences ionic currents in Kir channels (Fakler et al., 1994; McNicholas et al., 1994; Müllner et al., 2000).

While several Kir crystal (Nishida and MacKinnon, 2002; Pegan et al., 2005, 2006; Nishida et al., 2007; Tao et al., 2009; Clarke et al., 2010; Inanobe et al., 2010, 2011; Balana et al., 2011; Hansen et al., 2011; Whorton and MacKinnon, 2011, 2013; Bavro et al., 2012; Lee et al., 2016) and recently single-particle cryo-EM structures (Lee et al., 2017; Li et al., 2017; Martin et al., 2017; Wu et al., 2018) have been determined, the availability of open-state conformations is sparse and “mutant induced” (Clarke et al., 2010; Whorton and MacKinnon, 2011; Bavro et al., 2012). The most detailed structural information exists for G-protein–coupled Kir channels (also referred to as Kir3 or GIRK), which have been crystallized in the absence and presence of PIP_2_ and Na^+^, with the “activatory” mutant R201A with and without PIP_2_ (Whorton and MacKinnon, 2011) and in complex with Gβγ and Na^+^ (Whorton and MacKinnon, 2013).

G protein–coupled inwardly rectifying potassium (GIRK) channels are important mediators of neurotransmitters such as dopamine, acetylcholine, serotonin, or GABA. These modulatory neurotransmitters inhibit neuronal activity by stimulating G-protein–coupled receptors (G_i/o_ type) that couple to GIRK channels. Activation of Kir3 hyperpolarizes the membrane potential of neurons, reducing action potential firing (Hibino et al., 2010; Lüscher and Slesinger, 2010; Rifkin et al., 2017). Agonist-induced conductance of GIRK channels constitutes a classic mediator of inhibitory neurotransmission, while basal activity has been shown to be important for setting the level of excitability and resting membrane potential in neurons (Lüscher et al., 1997; Torrecilla et al., 2002; Chen and Johnston, 2005; Rishal et al., 2005; Wiser et al., 2006). Further, basal activity might play a role in working memory (Sanders et al., 2013). GIRK channels are implicated in the pathophysiology of Down’s syndrome, Parkinson’s disease, epilepsy, alcohol addiction, and ataxia (Lüscher and Slesinger, 2010). Mutation or deletion of residues in the selectivity filter can lead to rare diseases such as Keppen–Lubinsky syndrome (Gorlin et al., 2001; De Brasi et al., 2003; Masotti et al., 2015) and were additionally reported in several cases of aldosterone-producing adenomas (Choi et al., 2011; Scholl et al., 2012).

Gating regulation of GIRK channels by various ligands is complex and not fully understood. Electrophysiological studies showed that both Gβγ and PIP_2_ are important mediators for channel activation, with Na^+^ ions promoting further opening (Huang et al., 1998; Sui et al., 1998; Hibino et al., 2010). Huang et al. (1998) suggested that in *Xenopus laevis* oocytes, PIP_2_ alone can directly activate GIRK1/4 channels, and Gβγ stabilizes the interaction of PIP_2_ with the channel. It is known that GIRK channels have a lower affinity for PIP_2_ than the constitutively active Kir2 channels (Zhang et al., 1999; Du et al., 2004). While there is consensus about the essentiality of PIP_2_ for channel activation, contradictory results have been reported concerning the absolute requirement of Gβγ for gating of Kir3 channels. Wang et al. (2016b) used a planar lipid bilayer system to avoid the influence of endogenously occurring modulators of GIRK channel activation, which are present in expression systems like oocytes. The authors reported that the channel requires both Gβγ and PIP_2_ for a robust opening (Wang et al., 2016b). In contrast to this, Glaaser and Slesinger (2017) reported that in a liposome system with POPE/POPG lipids and intracellular Na^+^, PIP_2_ is sufficient for activation of GIRK2 channels. They proposed that activators like Gβγ and alcohol might work as positive allosteric modulators for activation by PIP_2_ .

**Figure 1.**
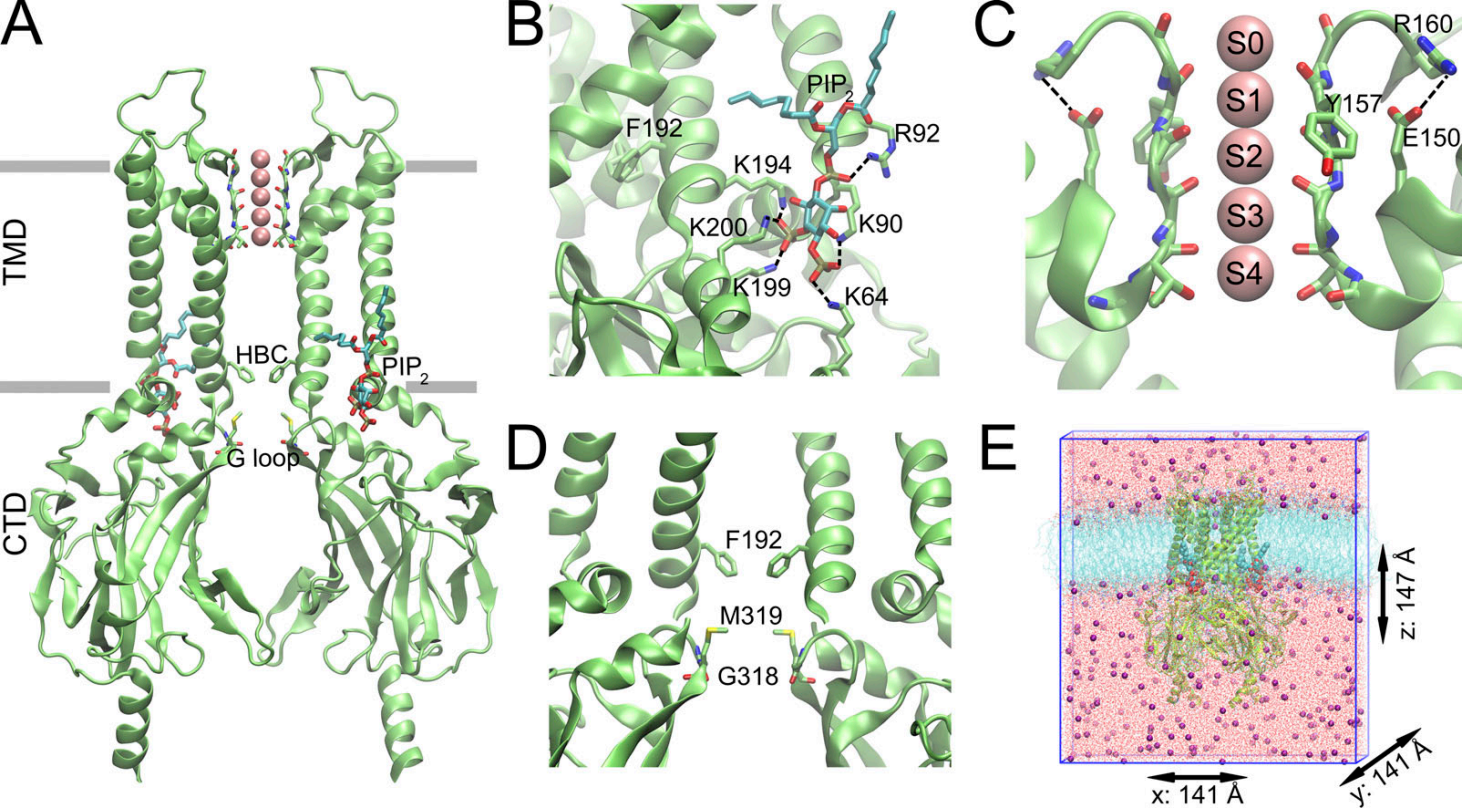
**Overview of GIRK2 structure. (A)** Two opposing subunits of the GIRK2 structure (PDB accession no. 3SYA) are shown; subunits in the front and back are hidden for clarity. The approximated membrane boundaries between the TMD and CTD are indicated as gray bars. **(B)** PIP_2_-binding site details: carbon atoms of the short-chain PIP_2_ are colored cyan, dashed lines indicate interactions with different basic amino acids; the F192 side chain, forming the narrowest part of the HBC gate, is also shown. **(C)** Details of the selectivity filter region with classical K^+^ ion–binding sites (S0–S4) shown as sticks; “bowstring” interactions between E150 and R160 are indicated with dashed lines. **(D)** HBC and G-loop gate regions formed by F192 and G318/M319 residues are shown as sticks, respectively. **(E)** Simulation box after equilibration: protein embedded in POPC membrane (cyan lines); water is shown in red (represented as lines); K^+^ ions (purple) and PIP_2_ (cyan lipid tail) are indicated as spheres.

In principal, atomic-resolution structures of GIRK channels in the absence and presence of gating modulators should enable detailed insights into the importance of the respective ligands and the induced conformational changes. Nevertheless, currently available x-ray structures of GIRK channels do not encompass the full conformational ensemble of functional states of the channel. In particular, lack of dynamics significantly limits our understanding of the molecular mechanism by which PIP_2_, Gβγ, and other ligands actually gate the channel. Contrary to expectations, based on functional data (Sui et al., 1998), cocrystallization of GIRK2 channels in complex with PIP_2_ and Gβγ only led to a “preopen”-state x-ray structure (Whorton and MacKinnon, 2013). Both the helix bundle crossing (HBC) gate and the G-loop gate (location shown in Fig. 1) reveal conformations that are largely identical with previously solved closed-state x-ray structures in presence of PIP_2_ (Whorton and MacKinnon, 2011). Only by cocrystallizing a constitutively active mutant (R201A) in the presence of PIP_2_ (but without Gβγ) were significant conformational changes at the gates observed. The R201A structure shows that rotation of the cytoplasmic domains (CTDs) and rotation and splaying apart of the inner transmembrane helices leads to a twofold symmetric structure (PDB accession no. 3SYQ) and opening of the G-loop gate to 15 Å between two subunits, while the distance between the narrower subunits amounts to 5.2 Å only (when measured between M319 side chains). The inner HBC gate opens up to 11 Å (wider pair), while the other two subunits remain at 5.4 Å. Considering a fourfold symmetry of the wider subunits, this would likely lead to gate conformations allowing the passage of hydrated K^+^ ions. Thus it was suggested that the R201A mutant structure might represent a conformation similar to a G-protein–activated one (Whorton and MacKinnon, 2011).

A general challenge for understanding ion channel gating on the molecular level is the often limited knowledge of functional states of structural conformations determined by x-ray, single-particle cryo-EM, or NMR approaches. MD simulations can provide key functional interpretation of experimentally determined structures. For example, MD simulations have been used successfully to provide atomistic insights into gating dynamics of ion channels (Biggin and Sansom, 2002; Beckstein et al., 2003; Grottesi et al., 2005; Domene et al., 2006; Mashl and Jakobsson, 2008; Delemotte et al., 2011, 2017; Amaral et al., 2012; Barber et al., 2012; Dryga et al., 2012; Jensen et al., 2012; Meng et al., 2012, 2016; Fowler et al., 2013; Linder et al., 2013, 2015; Kratochvil et al., 2016; Schewe et al., 2016; Heer et al., 2017; Starek et al., 2017 *Preprint*; Fernández-Mariño et al., 2018; Li et al., 2018; Jekhmane et al., 2019). Further, MD provided unprecedented insights into conduction (Bernèche and Roux, 2001; Delemotte et al., 2008; Furini and Domene, 2009; Jensen et al., 2010, 2013; Köpfer et al., 2014; Kasahara et al., 2016; Sumikama and Oiki, 2016; Kopec et al., 2018) and helped evaluate and assign functional states (Zhu and Hummer, 2012; Aryal et al., 2015; Trick et al., 2016; Rao et al., 2017, 2018) of such structures.

In this study, we performed MD simulations on the GIRK2 channel in complex with PIP_2_ (both gates annotated closed, based on distance measurements of the x-ray structures by the crystallographers; Whorton and MacKinnon, 2011), but in the absence of Na^+^, to investigate conformational dynamics of GIRK2 channels. MD simulations reveal that when embedded in a lipid membrane, relaxation of the crystal structure, together with side-chain flexibility, results in wetting of both gates. This leads to rapid, spontaneous opening and consequential K^+^ flux through the channel, revealing details of the ion permeation process.

## Materials and methods

Gromacs software version 5.1.2 (Abraham et al., 2015) was used to perform MD simulations. The Kir3.2 channel (PDB accession no. 3SYA; resolution 2.98 Å) was embedded in an equilibrated membrane using g_membed (Wolf et al., 2010), implemented in Gromacs version 4.6.7 (Hess et al., 2008), leading to a lipid bilayer consisting of 588 POPC (1-palmitoyl-2-oleoyl-sn-glycero-3-phosphocholine) lipids. We used Berger lipid parameters (Berger et al., 1997) for POPC and the amber99sb force field (Hornak et al., 2006) for the protein. We used the same PIP_2_ parameters as described in our previous work (Lee et al., 2016). K^+^ ions were placed in selectivity filter positions S0, S2, and S4, while single water molecules were put in positions S1 and S3 (Åqvist and Luzhkov, 2000). No Na^+^ ions were included in our simulations. The simulation box was solvated with 65,750 molecules of extended simple point charge model (SPC/E) water (Berendsen et al., 1987). After neutralizing with K^+^ ions, 150 mM KCl was added to the simulation system. For ion parameters, we used corrected monovalent Lennard–Jones parameters (Joung and Cheatham, 2008). The final system consisted of 249,910 atoms. For Lennard–Jones and electrostatic interactions, we used a cutoff of 1.0 nm. Long-range electrostatic interactions were calculated at every step with the particle-mesh Ewald algorithm (Essmann et al., 1995). Bonds were constrained using the LINCS algorithm (Hess et al., 1997), allowing an integration time step of 2 fs. Temperature was coupled to 310 K using the v-rescale thermostat (Bussi et al., 2007) and a coupling constant of 0.1 ps. The pressure was kept constant semi-isotropically at 1 bar by the Parrinello–Rahman (Parrinello and Rahman, 1981) barostat (τ = 2 ps). While restraining the protein atoms with a force constant of 1,000 kJ mol^−1^ nm^−2^ to their starting positions, we performed a steepest descent energy minimization of the simulation system, followed by 10 ns of NVT (constant-temperature, constant-volume ensemble) and 10 ns of NPT (constant-temperature, constant-pressure ensemble) equilibration runs.

Initially, 10 × 200 ns free MD simulations were performed, followed by 14 × 1 µs runs with an applied electric field of 20 or 40 mV nm^−1^ along the z axis of the simulation box (see Table 1). Considering a z-axis box length of ∼14.5 nm, this results in transmembrane potentials of 290 and 580 mV, respectively (Roux, 2008; Bjelkmar et al., 2009; Gumbart et al., 2012). The potential along the box, density profiles as function of box length, and area per lipid are shown in Fig. S1, A–C. In 5 out of 14 runs, the G-loop gate residues G318 and M319 were restrained with a force constant of 1,000 kJ mol^−1^ nm^−2^ to their initial positions. The protein was visualized using VMD (Humphrey et al., 1996) and Pymol (Schrodinger, 2015). The channel interior surface of the pore was calculated by the program HOLLOW (Ho and Gruswitz, 2008).

**Table 1.** Overview of MD data

MD run	Simulation time (µs)	Applied electric field (mV)	Ion sites occupied at starting state	HBC gate permeation events
200ns_run1	0.2	—	S2, S4	0
200ns_run2	0.2	—	S2, S4	0
200ns_run3	0.2	—	S2, S4	0
200ns_run4	0.2	—	S2, S4	0
200ns_run5	0.2	—	S2, S4	0
200ns_run6	0.2	—	S2, S4	1
200ns_run7	0.2	—	S2, S4	1
200ns_run8	0.2	—	S2, S4	1
200ns_run9	0.2	—	S2, S4	0
200ns_run10	0.2	—	S2, S4	0
				**Full SF permeation events**
1µs_run1	1	580	S1, S2, S4	30
1µs_run2	1	580	S1, S2, S4	9
1µs_run3	1	580	S1, S2, S4	0
1µs_run4	1	580	S2, S3	8
1µs_run5	1	580	S2, S3	27
1µs_run6	1	580	S2, S3	20
1µs_run7	1	580	S2, S3	15
1µs_run8^a^	1	580	S2, S3	24
1µs_run9^a^	1	580	S2, S3	19
1µs_run10^a^	1	580	S2, S3	27
1µs_run11	1	290	S2, S3	0
1µs_run12	1	290	S2, S3	19
1µs_run13^a^	1	290	S2, S3	27
1µs_run14^a^	1	290	S2, S3	3
Total number	16			228

10 replicas of 0.2-µs free MD simulations were performed using the equilibrated system of the crystal structure with bound PIP_2_ (PDB accession no. 3SYA). 14 1-µs simulations were performed with an applied electric field. 1µs_run1, 1µs_run2 and 1µs_run3 started from a snapshot at 50ns of 200ns_run8. All the other MD runs with applied electric field were initiated from a snapshot of 1µs_run1 (t = 400 ns), where the HBC gate reached a steady state of a relatively wide and solvated conformation (see Figs. S4 and S5 B). For all 1-µs simulations, either 40 mV nm^−1^ or 20 mV nm^−1^ was applied; this equals ∼580 mV or ∼290 mV, respectively, when calculated over the whole box (z axis; Roux, 2008; Bjelkmar et al., 2009; Gumbart et al., 2012).

a Harmonic restraints on G-loop gate–forming residues G318 and M319.

### Effective-biased potential of mean force (PMF^EB^) calculations

The PMF^EB^ (nomenclature as suggested previously by Miranda et al., 2018) for K^+^ in the channel pore was calculated for all unrestrained 1-µs trajectories with applied electric field. The Gromacs tool gmx trjconv was used to align the trajectories at the selectivity filter (sequence TTIGYG). The coordinates of potassium ions and protein were written out every 20 ps, resulting in 50,000 steps per simulation run. Along the membrane normal (= pore axis z), the area between the intracellular entrance of the channel and the end of the selectivity filter (SF) was cut into slices of 0.5-Å thickness. Potassium ions inside the channel pore were counted in each slice. Average numbers of resident potassium ions were plotted against the membrane normal. Based on these occupancies, the PMF^EB^ was determined using the following equation: G_PMF_(z) = −k_B_T ln *n*(z) (de Groot and Grubmüller, 2001).

### Calculation of the rotational angle

The relative rotation of the CTD with respect to the transmembrane domain (TMD) was determined as the torsional angle between two planes. This required four points of measurement: the center of mass of the TMD (point 1) and the CTD (point 2) and the center of mass of one subunit of the TMD (point 3) and the CTD (point 4). Points 1, 2, and 3 defined the first plane; points 1, 2, and 4 defined the second plane (see Fig. S2 A for an illustration). The torsional angles of the end states of all 1-µs runs were compared with the crystal structure (PDB accession no. 3SYA), which is shown in Table 3.

### Analysis of G-loop gate permeation events

To analyze all K^+^ ion permeation events at the G-loop gate, we extracted simulation steps with a K^+^ ion located inside a cylinder of 4-Å radius and 4-Å height within the center of mass of the G-loop gate–forming residues G318 and M319. Minimum distances between opposing gate-forming subunits were calculated by the Gromacs tool gmx mindist for every simulation snapshot, and the distance value of the narrower subunit pair was used to calculate an average of the distance during ion permeation. This value gives an approximation of the narrowness of the G-loop gate during ion permeation. The extent of the K^+^ ion solvation during permeation was determined using gmx select, by calculating the number of oxygen atoms (water molecules) within 3.5 Å of the K^+^ ion located in the G-loop gate.

### H-bond analysis

Hydrogen bond analysis was performed using the gmx hbond tool of Gromacs, which assesses hydrogen bonds based on the distance between donor and acceptor within 3.5 Å and an angle cutoff of 30 degrees.

### Online supplemental material

Fig. S1 shows the potential along the membrane normal and validation of membrane integrity. Fig. S2 is a schematic figure illustrating the points used to measure CTD rotation, including examples of PIP_2_-depleted runs. Fig. S3 shows RMSD values over time. Fig. S4 shows wetting of the inner pore of the channel with water molecules. Fig. S5 shows minimum distance calculations of opposing subunits at the G-loop gate. Fig. S6 shows minimum distance calculations of opposing subunits at the HBC gate. Fig. S7 shows an analysis of PIP_2_ dynamics. Fig. S8 illustrates the opening of the HBC gate caused by helix bending. Fig. S9 shows movement of individual ions through the channel as a function of simulation time. Fig. S10 shows water molecule traces in the selectivity filter. Fig. S11 shows how K^+^ ions are coordinated by carbonyl oxygens of G318 in different example snapshots. Table S1 lists an overview of all control runs. Video 1 visualizes the movement of a K^+^ ion through a narrow G-loop gate. Video 2 shows a K^+^ moving through the whole ion channel pore. Video 3 shows several K^+^ ions permeating the SF via a direct knock-on mechanism.

## Results

### Monitoring the dynamics of the GIRK2 structure (PIP_2_ bound)

**Figure 2.**
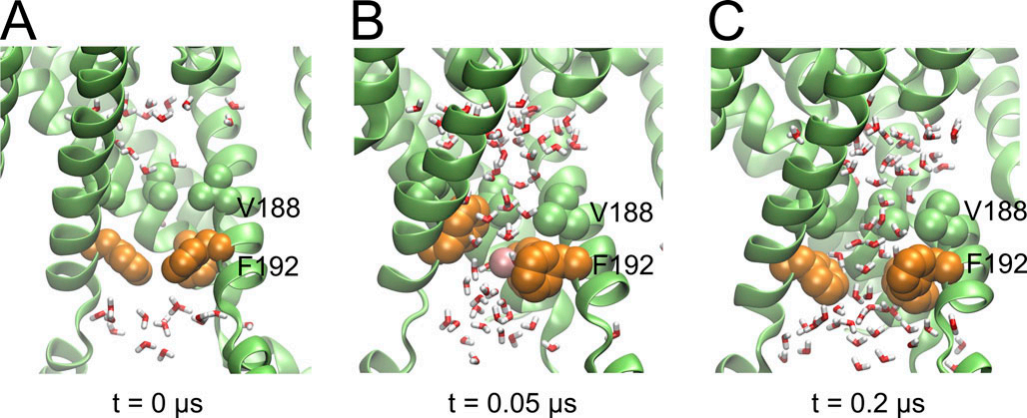
**Solvation and spontaneous K^+^ flux through the HBC gate.** Side view of the HBC gate region, with water molecules shown as sticks and a K^+^ ion (pink sphere) forming cation-π interactions. **(A)** Starting state of the simulation after equilibration of the simulation system. **(B)** Ion permeating the wetted HBC gate. **(C)** End state after 0.2 µs.

The general architecture of the GIRK2 channel is presented in Fig. 1. Ten replicas of 200-ns unbiased full atomistic MD simulations of the GIRK2 structure with bound PIP_2_, embedded in a POPC membrane, were performed. The stability of the different runs was assessed by calculating the RMSD of the backbone atoms, which converged to ∼2.5 Å (Fig. S3 A). At the beginning of these simulations, the cytoplasmic region, particularly at residue M319 (G-loop gate), and the HBC gate region, lined by hydrophobic residues F192 and V188, are dewetted (see Fig. 2 A). Within ∼40 ns, water molecules diffuse from the bulk into the inner cavity. In 4 out of 10 simulations, the cavity becomes solvated, while in 4 other runs, only partial wetting is observed. Two runs remain closed and thus dry on the level of the HBC gate (Fig. S4 shows solvation of the inner cavity over time). Successful solvation of the channel pore depended mainly on the side-chain conformation of M319 at the G-loop gate but also on the diameter of the HBC gate. Fig. S5 A shows minimum distance plots of opposing G-loop gate subunits, ranging between 2 and 10 Å in the 10 200-ns simulation replicas. For example, one pair of opposing subunits of 200ns_run4 (Fig. S5 A) was in close contact throughout the whole simulation, caused by tight interactions of the M319 side chains. This prevented solvation of the inner pore of the channel, as can be seen in Fig. S4. In contrast, 200ns_run8 showed a strong increase in the amount of inner cavity water (>80 water molecules; orange line in Fig. S4), which agrees with its wider G-loop gate, as plotted in Fig. S5 A.

**Figure 3.**
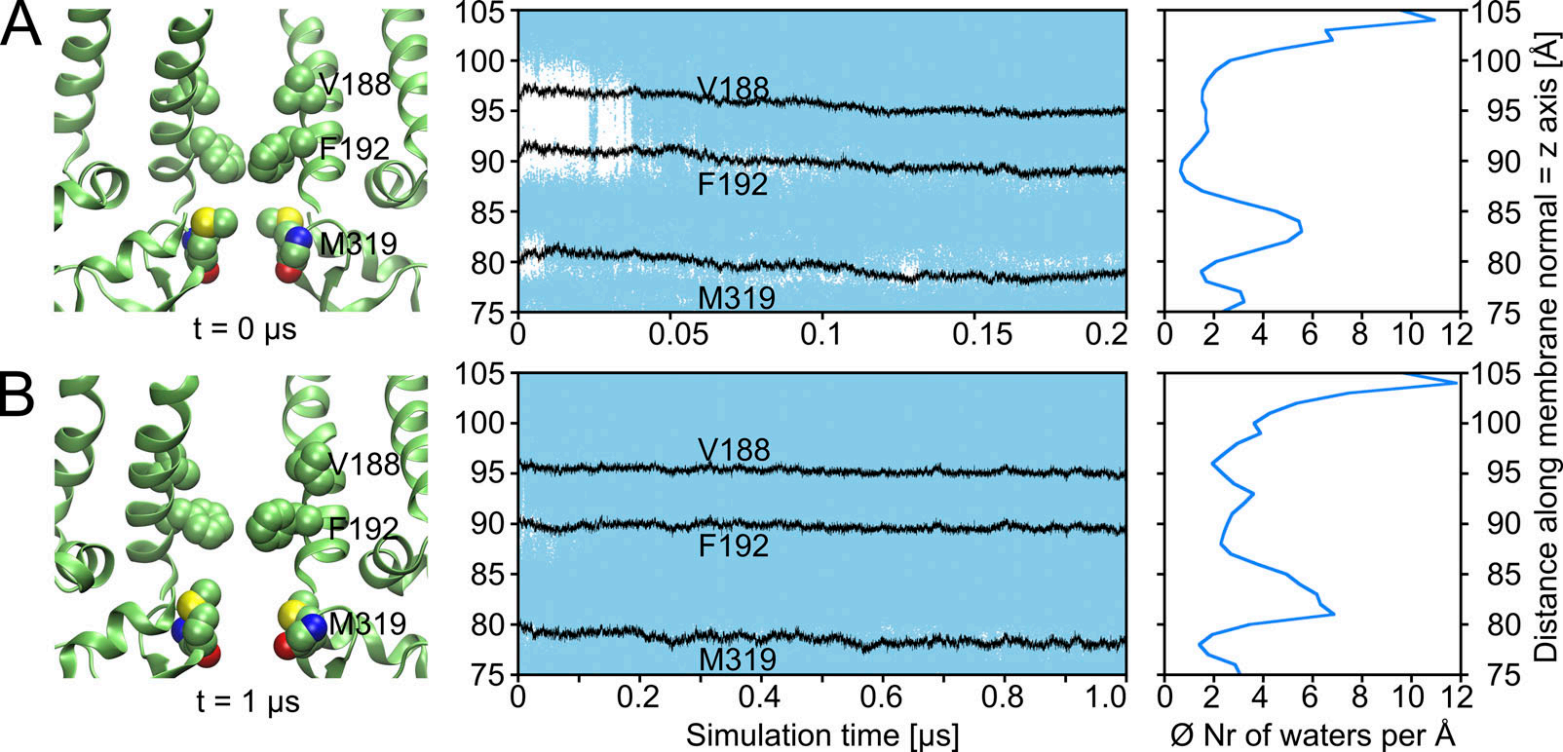
**Wetting of the G-loop gate and inner cavity. (A)** Left: Two subunits of the gate region in the crystal structure (PDB accession no. 3SYA); middle: water occupancy along the pore over simulation time; every blue dot represents a water molecule; for orientation, the centers of mass of the pore-facing hydrophobic residues M319, F192, and V188 are plotted in black. Right: Average occupancy (per 1 Å) of water molecules along the pore axis during the same 0.2-µs run (200ns_run8). **(B)** Example run (1µs_run1) with applied electric field; the left side shows the conformation of the gate region after 1 µs.

Surprisingly, in 3 out of 10 runs, K^+^ ions spontaneously transitioned from the bulk into the central cavity (see Table 1 under “HBC permeation events”), crossing a relatively narrow, partially wetted HBC gate region. Fig. 2 B shows an example of a K^+^ permeation event through the HBC gate (200ns_run8). As can be seen in Fig. S6 A, the minimal distance between opposing HBC-gate–forming F192 residues ranged from ∼3 to 10 Å. Fluctuations of the F192 side chain enable K^+^ ions to pass a partially solvated HBC region, possibly aided by favorable cation-π interactions (Fig. 2 B), as assessed by measuring the angle and distance between the F192 side chain and the K^+^ ion. Future studies with a higher level of theory will be needed to validate this result, since cation–π interactions are not well described by classical force fields (Lamoureux and Orabi, 2012). Nevertheless, our observations are consistent with previous simulations on the Kir3.1 chimera (Meng et al., 2016). The solvation process of this run is visualized in Fig. 3 A. The middle panel illustrates the pore axis (z axis) as a function of time. The plot covers the area between the G-loop gate and part of the inner pore above the hydrophobic pore facing residues F192 (HBC gate) and V188 (one turn above). At start the areas around M319, F192, and V188 are dewetted, as indicated by the white space. During the MD simulation, the area around the G-loop gate shows varying water occupancy, caused by side-chain flexibility of M319, indicated by changes in minimum distances of opposing subunits (Fig. S5 A). The hydrophobic area at the HBC gate starts to wet at ∼40 ns. While the region of V188 stays permanently solvated, the bulky hydrophobic side chain of F192 allows less solvation in this simulation (200ns_run8). This is also displayed in the right panel, where the average number of water molecules per Å along the membrane normal (= pore axis z) during the simulation is <1 in this particular region.

### Is PIP_2_ alone sufficient to activate GIRK2 channels?

Before assessing whether PIP_2_ alone might be sufficient to activate GIRK2 channels, we analyzed the dynamics of the bound PIP_2_ molecules, which were previously suggested to adopt a novel position upon opening (Lacin et al., 2017). As shown in Fig. S7 A, the bound PIP_2_ molecules are relatively stable, with RMSD values of ≤1.5 Å. Further, hydrogen bond formation between basic residues K194, K199, K200, K64, K90, R92, and PIP_2_ were assessed, as shown in Fig. S7 B. On average, persistent hydrogen bonds between these residues and PIP_2_ were observed in MD simulations without changes of the hydrogen-bonding patterns.

**Figure 4.**
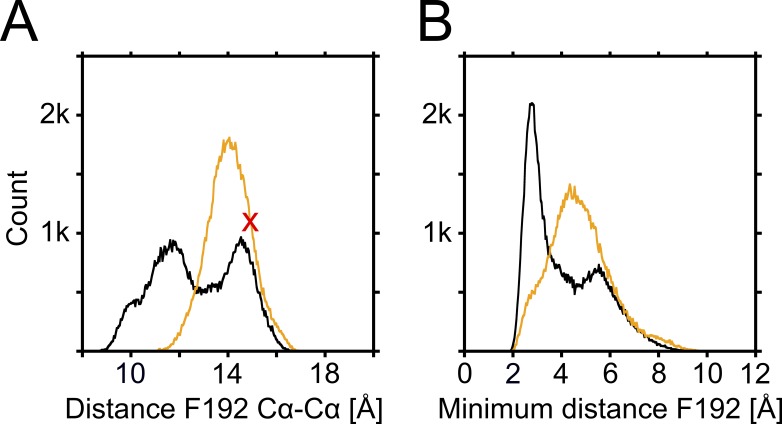
**Comparison of HBC gate distances of PIP_2_-bound and PIP_2_-depleted control simulations.** Combined F192 distance data of control simulations with (10 × 0.2 µs, orange color) or without (10 × 0.2 µs, black color) PIP_2_ bound. Table S1 gives an overview of all control simulations. **(A)** Distances between opposing Cα atoms of the HBC-gate–forming residue F192. The distance observed in the crystal structure (15.3 Å) is indicated by a red cross. **(B)** Minimum distances between opposing F192 residues. In both panels, only the narrower subunit pair was considered at each time step of all control runs.

Next, we performed 20 × 200–ns control simulations, with and without PIP_2_, starting from the same equilibrated states as above, but with an applied field of 40 mV nm^−1^. As illustrated in Fig. 4, A and B, there is a clear difference in the Cα and minimum distances at residue F192 (HBC gate) when comparing holo and apo systems. While the Cα distances in the crystal structure and after equilibration are already ∼15 Å wide (see red cross in Fig. 4 A), which might be considered “preopen,” the majority of channels close in the absence of PIP_2_, supporting the notion that PIP_2_ alone might indeed be sufficient for activation of GIRK2 channels.

### Conformational changes observed during microsecond-scale simulations

To gain in-depth insights into the ion-permeation mechanism of GIRK2, we applied an electric field of either 20 or 40 mV nm^−1^ (290 or 580 mV, respectively), as described previously (Roux, 2008; Gumbart et al., 2012; Meng et al., 2016; Kopec et al., 2018). To ensure membrane integrity at these nonphysiological membrane voltages, the area per lipid with and without applied field was compared (Fig. S1 C). Additionally, the density profiles along the box were calculated (Fig. S1 B).

In total, 14 × 1-µs simulations were performed. Table 1 gives an overview of all performed MD runs, except control simulations, which are shown in Table S1. The first three 1-µs runs started from a snapshot of the above described simulations (at 50 ns of 200ns_run8, after a K^+^ permeation event). In contrast to the short simulations, we observed significant and stable widening at the HBC gate during 1 µs (Fig. S8 shows an example MD: 1µs_run1). Thus, subsequent simulations (1µs_run4 to 1µs_run14) were starting from a snapshot of this run with a fully open HBC gate (t = 400 ns of 1µs_run1). The widening was caused by a bending motion of the transmembrane helix M2 (Fig. S8 B), which is in agreement with published crystallographic (Bavro et al., 2012) and MD data (Rosenhouse-Dantsker and Logothetis, 2006; Meng et al., 2016; Lacin et al., 2017). Fig. S8 C illustrates an increased channel interior surface, especially in the area between the G-loop gate and residue V188 (one turn above the HBC gate). Besides the bending of helix M2, a slight outward rotation of the pore-facing residues F192 and V188 (Fig. S8 B) occurred, aiding channel wetting. Fig. 3 B illustrates the water occupancy in a 1-µs run. Opening of the HBC gate during this simulation allowed permanent wetting of the pore. This is also visible in the increased average number of water molecules per 1 Å along the pore axis (z axis), which can be seen by comparing the plots on the right side of Fig. 3, A and B, at residue F192.

**Figure 5.**
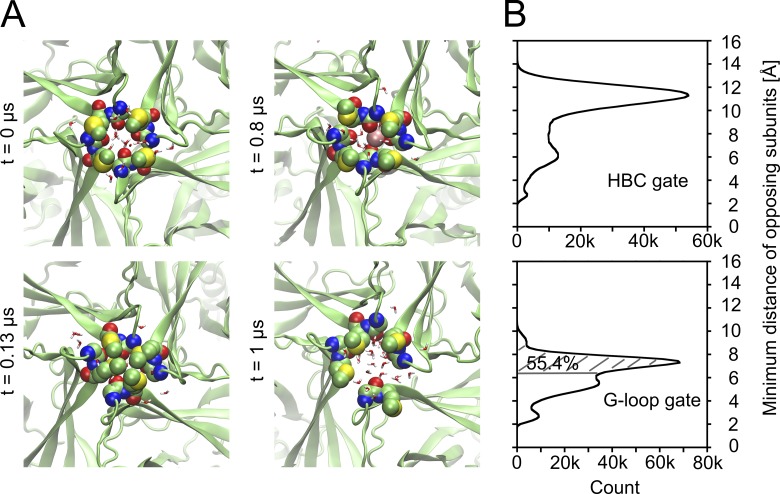
**G-loop gate conformations and minimum distances. (A)** Representative snapshots of the G-loop area at different time steps showing M319 and G318 as spheres and water molecules within 4 Å as sticks; a K^+^ ion is shown as a pink sphere. **(B)** Histograms with minimum distances between opposing subunits at the HBC gate (upper plot) and the G-loop gate (lower plot); residues A316 to T320 were included for analysis of the G-loop gate. The G-loop gate was wider than the average pore diameter during ion permeation (6.4 Å; see Table 2) in 55.4% of all simulation steps, as indicated by the hatched area. 11-µs data were included (unrestrained runs) for both plots.

The average number of water molecules at residue M319 is very similar in both runs displayed in Fig. 3, A and B. Contrary to the HBC gate, there was no significant widening of the G-loop gate in the longer runs. The minimum distances of opposing G-loops vary from ∼3 to 11 Å, as described above (shown in Fig. S5 B). Again, this was caused by flexible methionine side chains, leading to alternating wetting/dewetting and the lowest average water occupancy at this gate. Selected snapshots presenting different open and closed states at the G-loop gate are illustrated in Fig. 5 A. A combined analysis of gate minimum distances of all unrestrained runs presented in Table 1 is shown as histograms in Fig. 5 B. Comparing both histograms reveals that the G-loop gate is remarkably narrower than the HBC gate during MD simulations. Video 1 shows an example how an ion permeates the G-loop gate in a narrow conformation.

### Conduction mechanism in GIRK channels

**Figure 6.**
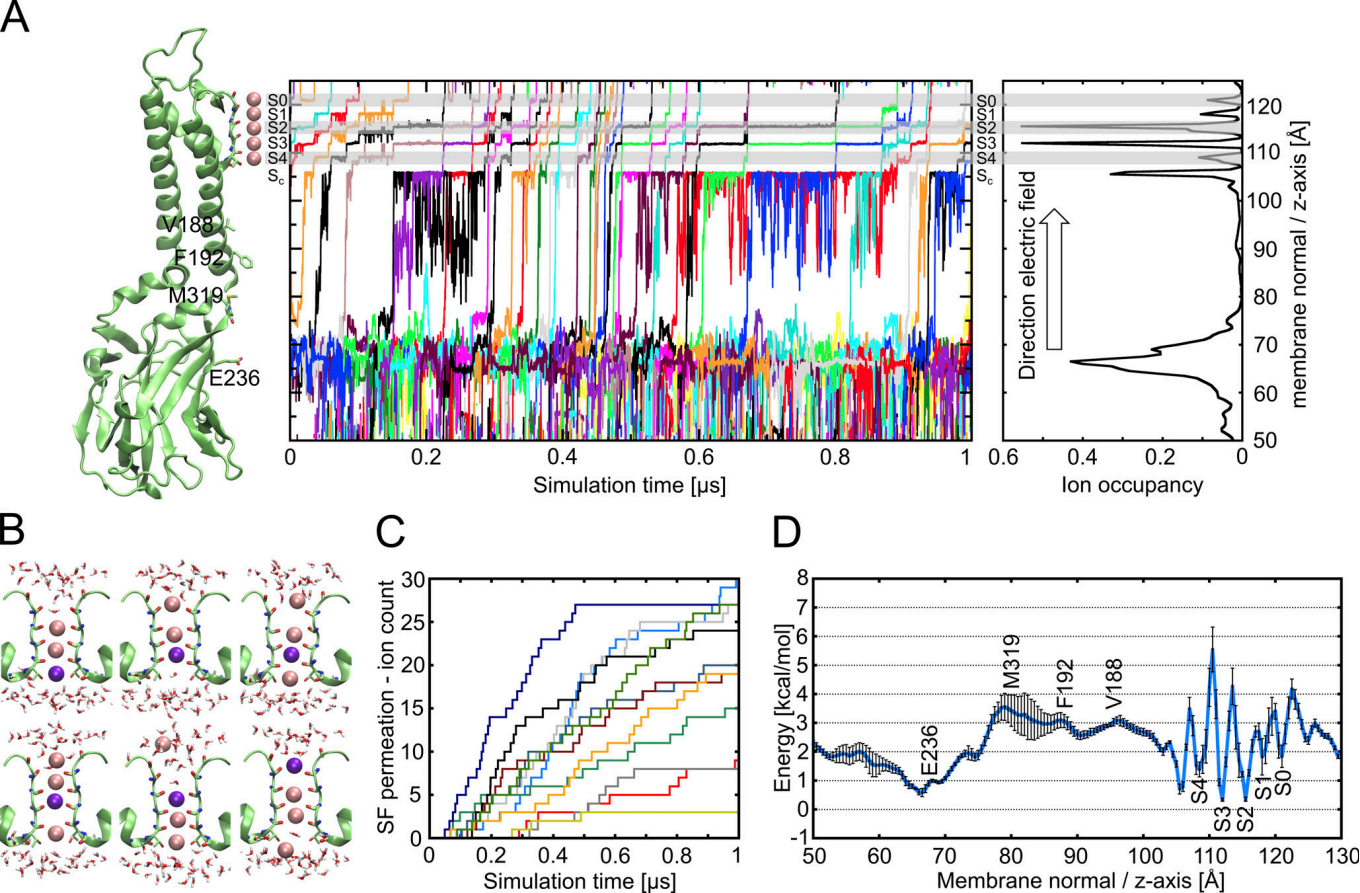
**Conduction mechanism in PIP_2_–GIRK2. (A)** Left: Cartoon representation of a single Kir3.2 subunit, with key residues shown as sticks. Middle: K^+^ ion movement along the pore axis (z axis) as a function of time, represented by differently colored lines. Right: Ion occupancy, calculated every 0.5 Å along the pore axis. **(B)** Representative snapshots of an ion traversing the SF of Kir3.2 with the permeating ion colored in dark purple. **(C)** Cumulative ion flux over simulation time; 12 runs with different simulation conditions included (290 or 580 mV, restrained or unrestrained G-loop); each run is colored differently. **(D)** PMF^EB^ derived from the K^+^ occupancy along the pore (unrestrained runs), with black error bars (SD).

The conditions used for the MD runs with applied electric field are summarized in Table 1. To test the influence of G-loop gate flexibility and diameter on K^+^ ion permeation, we additionally performed simulations with restraints. Harmonic restraints (force constant of 1,000 kJ mol^−1^ nm^−2^ on G318 and M319 in an open conformation) were applied for five runs, effectively restraining the gate diameter to 7.5 Å between opposing subunit pairs. Restrained runs are marked (Tables 1, 2, and 3). Ion flux through the unrestrained channel is illustrated in Fig. 6 A, using the same example run (1µs_run1) as presented in Fig. 3 B. Runs 2 to 14 are shown in Fig. S9. The colored lines represent different ions moving along the pore axis as a function of time. The right panel of Fig. 6 A shows that K^+^ ions are highly localized near the negative charges that underlie rectification (E236; Yang et al., 1995; Kubo and Murata, 2001; Pegan et al., 2005) in the CTD, as well as around residue T153, at the entrance of the selectivity filter (site “S_c_”), and at sites S2 and S3. From the intracellular side, ions first approach the CTD by free diffusion, with no preferred path, until they are coordinated by E236. Ions then transition to the region of the G-loop gate, where they single file. Depending on the M319 side-chain conformation, ion flux is possible or halted (Fig. 5 A). The ions pass the opened HBC gate in a fully solvated state (see Fig. 3 B, middle panel around F192 to see solvation of the gate; Fig. S8 A for widening), but in single file, to reach the inner cavity, as seen in Video 2. The inner cavity is occupied by up to two K^+^ ions. The ions permeate the selectivity filter (Fig. 6 B), formed by residues T154–G158, in a fully dehydrated manner, consistent with the previously described “direct Coulomb knock-on” mechanism (Köpfer et al., 2014; Kopec et al., 2018). Water molecule traces for all runs are shown in Fig. S10. The ion entering S4 rapidly pushes the ion from site S3 via a direct knock-on upward to S2, leading to a rapid exit (within several ns) of the ion initially located at site S2 to the extracellular side, via sites S1 and S0 (Fig. 6 B). Residence times for K^+^ ions in sites S3 and S2 varied from 5 to 130 ns. Although an original “classical” setup of alternating ion–water–ion in the selectivity filter (Åqvist and Luzhkov, 2000) was used for the initial simulation setup, conduction in all observed events occurs exclusively in a dehydrated manner via direct knock-on. This is illustrated in Video 3, which includes the time range from 418 to 480 ns of run 1 (1µs_run1). Within this time, five ions are moving through the selectivity filter.

**Table 2.** Analysis of the G-loop gate during ion permeation events

MD run	Ø minimum distances between opposing G-loop gate subunits when ions pass the gate (Å)	Ø number of coordinating water molecules
1µs_run1	5.90	4.73
1µs_run2	6.47	5.66
1µs_run3	6.75	5.64
1µs_run4	6.65	5.54
1µs_run5	6.40	5.34
1µs_run6	6.37	5.24
1µs_run7	6.84	5.71
1µs_run8^a^	7.19	5.69
1µs_run9^a^	7.13	5.88
1µs_run10^a^	7.18	5.77
1µs_run11	6.05	4.68
1µs_run12	6.05	5.47
1µs_run13^a^	7.10	5.93
1µs_run14^a^	7.14	5.78

For this analysis, only those MD steps with a K^+^ ion within a certain area of the G-loop gate (cylinder with a radius and height of 4 Å placed at the center of mass of the G-loop gate forming residues G318 and M319 of all four subunits), were extracted from the MD runs and used for analysis of minimum distances and solvation. This leads to an average pore diameter of 6.4 Å (unrestrained runs) during ion permeation. For determining the extent of solvation, all oxygen atoms of water molecules within 3.5 Å of the K^+^ ion were counted. The optimal ion coordination number in liquid water was estimated to be 6.9–7 within 3.5 Å (Rowley and Roux, 2012). The lower values shown in the table indicate that the ions permeated in a partially solvated manner, which was facilitated by coordination with the carbonyl oxygens of G318 residues (see Figs. 5 A and S11).

a Harmonic restraints on G-loop gate–forming residues G318 and M319.

**Table 3.** Rotation of the CTD with respect to the TMD: MD end states versus crystal structure

MD run	Angle (°)
1µs_run1	−3.89
1µs_run2	−3.90
1µs_run3	−0.09
1µs_run4	−4.31
1µs_run5	−4.46
1µs_run6	−2.88
1µs_run7	−4.46
1µs_run8^a^	−2.21
1µs_run9^a^	−4.66
1µs_run10^a^	−4.32
1µs_run11	−2.91
1µs_run12	−4.29
1µs_run13^a^	−5.60
1µs_run14^a^	−1.90

End states of all 1-µs MD runs vs crystal structure (PDB accession no. 3SYA); the negative values indicate a counterclockwise rotation (viewed from top of the protein).

a Harmonic restraints on G-loop gate–forming residues G318 and M319.

Overall, within 14 µs, we observed 228 individual K^+^ ion permeations. Permeation events occurred at rates between 3 and 30 ions per microsecond (Fig. 6 C), while two runs displayed no conductance. With an applied transmembrane field of 290 or 580 mV, this corresponds to conductance rates of 2.3–14.9 pS. Remarkably, this is similar as the experimentally measured single-channel conductance in GIRK2 acetylcholine-activated inward rectifier current (K_ACh_) channels in oocytes of 30 pS (Kofuji et al., 1995). K^+^ ion distribution averaged over unrestrained simulations reveals energy barriers at the G-loop gate and the SF (Fig. 6 D). With ∼2 kcal mol^−1^, the barrier of the G-loop gate is very low, and no noticeable barrier is observed at the HBC region, suggesting that these locations will not function as significant rate-limiting steps for K^+^ ion permeation. Surprisingly, the energy barriers at the SF (∼4 kcal mol^−1^) are noticeably higher than at the G-loop gate. This suggests that the SF itself will provide a rate-limiting step for K^+^ conduction through the open GIRK2 channel. Nevertheless, in absolute terms, the observed energy barrier at the SF is only slightly higher than what would be expected for diffusion limited ion flux (Bernèche and Roux, 2001) and hence will not preclude high K^+^ conductance through the channel. Comparison of average permeation rates of unrestrained (14.2 ± 10.8 events per microsecond) with restrained (20 ± 10 events per microsecond) suggests rather minor effects of G-loop gate diameter and flexibility on conductance.

### Distance at the highly conserved Y157 in the SF correlates with flickering

**Figure 7.**
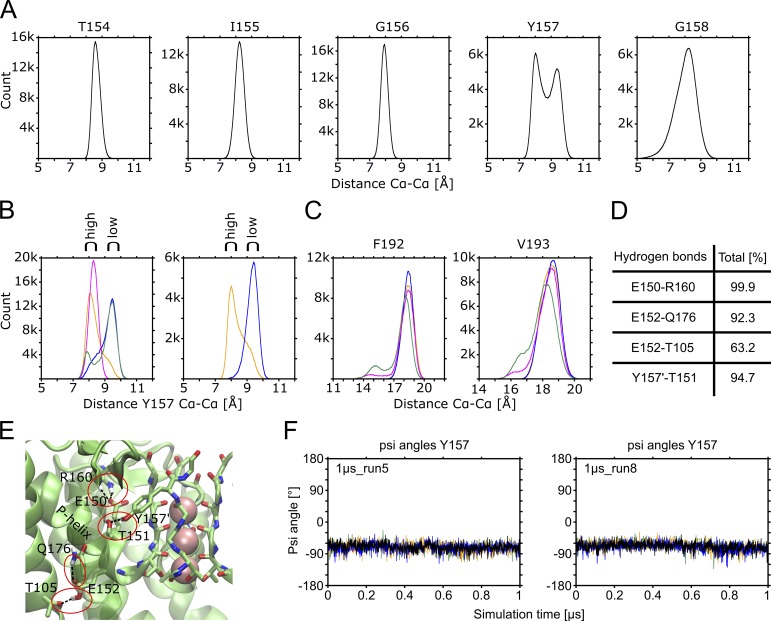
**Conformational analysis of the SF region. (A)** Distances between opposing Cα atoms of SF-forming amino acids (combined data of all 14 × 1-µs runs). **(B)** Left plot: Comparison of distance data of opposing Y157 Cα atoms between clusters of MD runs with high (magenta, 1µs_run1 + 1µs_run5 [40 mV nm^−1^]; orange, 1µs_run12 + 1µs_run13 [20 mV nm^−1^]) or low (green, 1µs_run2 + 1µs_run4 [40 mV nm^−1^]; blue, 1µs_run11 + 1µs_run14 [20 mV nm^−1^]) conductance. Right plot: To analyze temporary conductance fluctuations, snippets of phases with high (orange) or low (blue) conductance of the MD runs 1µs_run5 (0.2–0.5 µs = high; 0.7–1 µs = low) and 1µs_run8 (0–0.4 µs = high; 0.6–1 µs = low) are clustered. See also Fig. S9. **(C)** Distances between opposing Cα atoms of residues at the HBC gate (F192 and V193). The runs were clustered and colored the same way as in the left plot of B. **(D)** Analysis of hydrogen bonds in the SF area (combined data of all 14 × 1-µs runs). **(E)** Visualization of the hydrogen bonds in the SF area. The front subunit is hidden for better visibility. **(F)** Psi angles of residue Y157 as a function of time for two MD runs with phases of high and low conductance. Each plot shows the psi angles of all four subunits (colored in black, blue, green, and orange).

Notably, the conductance rates in the different trajectories fluctuated widely, with two runs displaying no conductance at all (Table 1). To investigate which structural features might be responsible for low-conductive and nonconductive behavior, we monitored filter fluctuations, as described previously for K channel of streptomyces A (KcsA; Heer et al., 2017; Li et al., 2018). Fig. 7 A shows histograms of Cα distance measurements for all opposing SF residues. Since Y157 reveals two clusters, we analyzed this residue in more detail. Separation of conductive and nonconductive runs reveals that the Cα distances between opposite Y157 residues display ∼1 Å longer distances in the nonconductive runs (Fig. 7 B, left side). To further evaluate whether this increased distance indeed correlates with reduced ion flux, we extracted in total 700 ns from different nonconductive phases of runs 5 and 8, and in total 700 ns from high-conductive phases of the same runs (see Fig. S9), and plotted the distance histograms, as illustrated in Fig. 7 B (right side). These analyses suggest that the Y157 distance correlates with ion flux. It has previously been suggested that the selectivity filter is allosterically tuned by opening of the inner gate in KcsA (Heer et al., 2017; Li et al., 2018). Thus, we further analyzed if the gate distances at the level of the narrowest part of the HBC gate (residue F192), as well as the position corresponding to the gate in KcsA (T112 = V193 in GIRK2), are correlated with the observed selectivity filter changes. As shown in Fig. 7 C, the distances at both residues do not differ between high- and low-conductive states, suggesting that Kir channel flickering stems from a different mechanism. In agreement with this notion, carbonyl-oxygen flips of Y157 are not observed, irrespective of the conductance state (Fig. 7 F). This is perhaps not surprising, given the fact that Kir channels do not undergo C-type inactivation (McCoy and Nimigean, 2012). Filter stability mainly stems from a persistent salt bridge between residues E150 and R160 and additional hydrogen bonds, as illustrated in Fig. 7, D and E.

### Role of the G-loop gate for ion permeation

As described above, we observed a relatively narrow G-loop gate with alternating wetted and dewetted phases throughout our simulations, mainly caused by flexible side chains of M319. To analyze this in more detail, we monitored the G-loop gate distances during permeation events. All MD steps with K^+^ ions permeating the G-loop gate were extracted and the minimal gate distances during ion flux events monitored. The observed average minimal distances range from 5.9 to 6.8 Å (listed in Table 2) during unrestrained runs, leading to an average of 6.4 Å. Surprisingly, this value is smaller than the first hydration shell of a K^+^ ion. Our simulations reveal that oxygen atoms of G318 participate in ion coordination, partially mimicking the water shell, thereby allowing K^+^ ions to pass the G-loop gate at average distances lower than 8 Å. Fig. S11 illustrates example snapshots of K^+^ ions coordinated by carbonyl oxygens of G318. To estimate the extent of solvation during ion permeation, we calculated the number of oxygen atoms of water molecules within 3.5 Å of K^+^ ions moving through the G-loop gate. Table 2 lists the number of K^+^ ion coordinating water molecules for all 1-µs runs. The values show that the ions permeate the G-loop gate in a partially solvated manner.

### Rotation of the CTD

The rotation of the CTD with respect to its transmembrane domain was suggested to play an important role for gating of Kir channels (Clarke et al., 2010; Whorton and MacKinnon, 2011, 2013; Bavro et al., 2012; Wang et al., 2012, 2016a; Linder et al., 2015); however, no conclusive decision about the correlation could be reached so far. Thus, we analyzed the rotational angles of the CTD, as detailed in Materials and methods. As seen in Table 3, a counterclockwise rotation (viewed from top of the protein) up to 5.6° was observed in all runs, consistent with crystallographic (Whorton and MacKinnon, 2011) and FRET studies (Wang et al., 2012, 2016a). It is currently unclear whether rotation of the CTD is directly linked to channel gating. Thus, we extracted rotation angles from the 10 × 200–ns PIP_2_ depleted control simulations, which provide a good starting set to analyze if channel closure, as observed in our PIP_2_-depleted runs, could be correlated with CTD rotation. The CTD rotation over time of four representative closing runs is shown in Fig. S2 B. Comparison of the different runs reveals rotation angles ranging from −8° to 6°, suggesting lack of coupling between CTD rotation and gating of GIRK2 channels.

## Discussion

### Water-mediated gating changes of the PIP_2_-bound GIRK2 structure

Gating of GIRK channels is a complex process that, despite years of intense functional, structural, and computational studies, is far from completely understood. Even though x-ray complexes in the presence of the main activating modulators PIP_2_ (Whorton and MacKinnon, 2011) or a combination of PIP2, Gβγ, and Na^+^ (Whorton and MacKinnon, 2013) have been solved, these structures have been defined essentially closed based on distance measurements at the gates. MD simulations are very useful to study dynamic behavior of ion channels. Thus, here we performed a total of 18 µs MD simulations on the GIRK2 channel with bound PIP_2_, and 2 µs after depletion of PIP_2_, to investigate channel gating.

Unexpectedly, simulations reveal that in the absence of crystallographic constraints, PIP_2_-bound GIRK2 channels are conductive, despite the lack of Gβγ, considered critical for robust channel opening (Huang et al., 1998; Sui et al., 1998; Hibino et al., 2010; Wang et al., 2016b). Relaxing of the crystal structure in a more native-like membrane environment led to rapid “wetting” of both the G-loop and the HBC gates, followed by K^+^ ion permeation. Conductance rates observed in the simulations (up to 14.9 pS) are in good agreement with experiment (30 pS; Kofuji et al., 1995), supporting the notion that the gates are in a “fully open, conductive” conformation. Conformational changes at the HBC gate, supported by wetting of the gates, led to a rotation and splaying apart of the inner transmembrane helices (Fig. S8 B) in a similar manner, as seen in the PIP_2_-bound R201A–GIRK mutant channel. This is the only “mutant-induced” open state structure available for this family of inward rectifier K^+^ channels (Whorton and MacKinnon, 2011; PDB accession no. 3SYQ). The R201A mutant is located in a linker between the transmembrane and the CTD. Importantly, the opening of the HBC gate in our simulations leads to similar values (∼9–13 Å; Fig. S8 A) as observed in this presumably open structure (PDB accession no. 3SYQ). Once reached, this open state remains stable throughout all simulations. However, a clear difference is observed in the behavior of the G-loop gate. The twofold symmetric R201A-PIP_2_ x-ray structure shows a distance of 14.2 Å between its wider subunit pair (8.6 Å between its narrower pair), measured between oxygens of G318. Contrary to the HBC gate, the G-loop gate is highly flexible during our 1-µs simulations, consistent with previous x-ray studies (Pegan et al., 2005). Distances fluctuated between 2 and 11 Å (Fig. S5) due to conformational flexibility of the M319 side chains, rapidly switching between permeable and temporary impermeable states (Fig. 5 A and Video 1). The dilations in the G-loop gate are smaller than the 14 Å observed in the R201A–PIP_2_ x-ray structure, suggesting that the conformational changes of the R201A mutant may influence the G-loop gate in a different way than seen in our wild-type structure simulations.

### Unique role of the G-loop gate

An unexpected finding of our study concerned the fact that the carbonyl oxygen of glycine residue 318, located in the G-loop gate, participated in K^+^ coordination, partially mimicking the water shell (Fig. S11 and Video 1). This explains why effective pore diameters (Ø 5.9–7.19 Å; see Table 2) smaller than the size of a hydrated K^+^ ion (∼8 Å) enabled unrestricted K^+^ movement through this region. This is supported by the estimated free energies of permeation as shown in Fig. 6 D, revealing no significant barrier at the G-loop region, suggesting that the structure is functionally open. Our simulations suggest that in contrast to the HBC gate, the G-loop gate does not follow the classical hydrophobic gating mechanism (Beckstein and Sansom, 2006; Treptow and Tarek, 2006; Aryal et al., 2015; Jia et al., 2018) to control the ion diffusion process but rather lowers the energetic cost for ion dehydration in a similar way as in the selectivity filter of ion channels.

### Why is the Kir3.2 channel conductive despite the absence of Gβγ?

A puzzling question is why the Kir3.2 channel is open and conductive in our simulations when only PIP_2_ is bound, which seems in conflict with a study by Wang et al. (2016b). The authors used a planar lipid bilayer system to avoid the problem of endogenously present modulators of GIRK channels and to be able to control the experimental conditions more directly. They report that both PIP_2_ and Gβγ were absolutely required for a robust GIRK2 channel opening and a large K^+^ current (Wang et al., 2016b). On the other hand, flux assays with liposomes using POPE/POPG lipids and intracellular sodium suggested that PIP_2_ is sufficient for GIRK2 activation (Glaaser and Slesinger, 2017). The authors show that addition of alcohol and cholesterol leads to activation of GIRK2 in the presence of PIP_2_. They further recognized that these activators lead to an increase in PIP_2_ affinity, which they already described previously by electrophysiological experiments in human embryonic kidney cells (Bodhinathan and Slesinger, 2013). The authors further propose that PIP_2_ might be the agonist of GIRK channels, while activators like Gβγ and alcohol might function as positive allosteric modulators (Glaaser and Slesinger, 2017). This explanation is consistent with earlier electrophysiological experiments performed with oocytes, suggesting that PIP_2_ activation is stabilized by Gβγ (Huang et al., 1998). Huang et al. proposed that the low basal activity of GIRK channels arises from their low binding affinity for PIP_2_ in the absence of Gβγ. In agreement with this notion, the strength of the PIP_2_ channel interaction has been shown to not only influence the level of regulation of Kir channels by different modulators (Du et al., 2004), but also basal channel activity. It is known that Kir2.x channels, which show a high affinity for PIP_2_, are constitutively active (Hibino et al., 2010), while GIRK channels have relatively low PIP_2_ affinities (Zhang et al., 1999; Zhou et al., 2001). Perhaps the simplest explanation for opening of the channel in our simulations could be that conditions in the x-ray experiment led to a GIRK2 structure, where PIP_2_ molecules are already bound in a high-affinity conformation, despite the absence of the Gβγ subunits. In agreement with this reasoning, PIP_2_ binding sites are identical in the solved x-ray structures in the absence (PDB accession no. 3SYA) and presence (PDB accession no. 4KFM) of Gβγ. Moreover, previous simulations on the chimeric GIRK1 mutant M170P enabled successful opening of the channel without noticeable changes in the PIP_2_-binding mode (Meng et al., 2016). In contrast, recent simulations on the K200Y mutant of the GIRK2-PIP_2_/Gβγ (4KFM) proposed a moderate change in the PIP_2_ channel interactions during channel opening, while leading to the same opening motions in the transmembrane gate (Lacin et al., 2017). Unfortunately, in currently available GIRK structures containing PIP_2_, electron densities are missing for most positively charged residues that interact with PIP_2_. Thus, additional studies, preferably including x-ray structures with higher resolution at the PIP_2_ binding site, will be needed to clarify this issue.

### Mechanism of K^+^ conduction in GIRK channels

While ion translocation mechanisms through the selectivity filter of KcsA and different voltage-gated K^+^ (K_v_) channels have been studied extensively (Bernèche and Roux, 2001; Furini and Domene, 2009; Jensen et al., 2010, 2013; Köpfer et al., 2014; Kasahara et al., 2016; Sumikama and Oiki, 2016; Kopec et al., 2018), very limited information exists on the conduction mechanism of inward-rectifier K^+^ channels (Meng et al., 2016). In this study, we present the first detailed insights into the mechanism of K^+^ conduction through the inward-rectifier K^+^ channel Kir3.2. Studies on K_v_ channels have shown that ion translocation involves concerted movement of ions through the selectivity filter, with K^+^ ions separated by water molecules (Åqvist and Luzhkov, 2000; Bernèche and Roux, 2001). In 2009, Furini and Domene proposed that less concerted transitions with site vacancies may be energetically possible as well (Furini and Domene, 2009). Recent landmark studies, based on exhaustive sampling MD simulations, suggested that conductance in K_v_ channels and KcsA is governed by a direct knock-on mechanism (Köpfer et al., 2014; Kopec et al., 2018), as we now observed for Kir3.2 as well. Except for the starting configuration, K^+^ ions permeate the channel via a direct knock-on mechanism (fully dehydrated; Figs. S9 and S10), where an ion entering at site S4 pushes ions preferentially binding to sites S3 and S2 upward, leading to the exit of the outermost ion on the extracellular side (Fig. 6 B and Videos 2 and 3). Importantly, this mechanism is in agreement with ion occupancies observed in KirBac channel (bacterial homolog of Kir channels) crystal structures, revealing ion occupancy of neighboring binding sites, without alternating water molecules in some structures (Clarke et al., 2010).

There is a general lack of consensus concerning “direct” knock-on, as observed in our simulations, versus “soft” knock-on, as described previously in the majority of simulations (Åqvist and Luzhkov, 2000; Bernèche and Roux, 2001; Domene and Sansom, 2003; Khalili-Araghi et al., 2006; Furini and Domene, 2009; Jensen et al., 2010, 2013; Ceccarini et al., 2012; Fowler et al., 2013; Kasahara et al., 2013). These discrepancies mainly stem from the use of different force fields, which seem to favor different conductance mechanisms. Unfortunately, attempts to experimentally distinguish between the different conductance mechanisms are inconclusive. While recent two-dimensional infrared spectroscopy studies by Kratochvil et al. (2016, 2017) were interpreted in favor of the water-mediated ion flux mechanism, this was later questioned by Kopec et al. (2018), who reported that the experiments are equally consistent with a direct knock-on mechanism. Another recent study by Langan et al. (2018), using single-wavelength anomalous dispersion x-ray diffraction data, lends further support for the direct Coulomb knock-on hypothesis. Ultimately, this issue will require further experimental analyses.

### Role of selectivity filter for GIRK2 gating

Interestingly, our simulations suggest that the highest energy barrier for K^+^ ion movement is located in the selectivity filter (∼4 kcal mol^−1^; Fig. 6 D). This prediction is in agreement with previous x-ray footprinting studies on KirBac3.1 (Gupta et al., 2010), as well as the identification of activating mutations found in the selectivity filter region (Paynter et al., 2010).

The influence of the SF on conductance and gating in K^+^ channels has been widely studied previously (McCoy and Nimigean, 2012; Ostmeyer et al., 2013; Liu et al., 2015; Conti et al., 2016; Matulef et al., 2016; Schewe et al., 2016; Cuello et al., 2017; Pau et al., 2017; Labro et al., 2018). For example, recent studies by Heer et al. (2017) and Li et al. (2018) on KcsA revealed an allosteric cross talk between the selectivity filter and the activation gate. It has been shown that C-type inactivation, which leads to filter narrowing in these channels, correlates with the diameter of the activation gate. Mutating a highly conserved salt bridge behind the SF, the so-called bowstring in Kir3.1/Kir3.4 channels, has also been shown to perturb filter conformation and selectivity, as well as influences gating regulation by Gβγ (Claydon et al., 2003). In agreement with these filter-gating observations, our Kir3.2 simulations reveal high- and low-conductance phases on the microsecond timescale. In an attempt to correlate these phases with conformational changes in the filter, distance analyses of all SF residues were performed (Fig. 7). Interestingly, these analyses revealed a dilation at the highly conserved Y157 position, which together with G158 forms site S1 in the SF. The importance of this site for conformational changes during C-type inactivation has previously been proposed (Hoshi and Armstrong, 2013; Armstrong and Hoshi, 2014). Further, a recent x-ray structure of Kv1.2-2.1 chimera in a putatively inactive conformation reveals distortions at this position, albeit again leading to narrowing of the SF at this site (Pau et al., 2017). This might reflect an important difference between K_v_ and Kir channels, which in contrast to the former lack C-type inactivation (McCoy and Nimigean, 2012). Further support for different filter-gating mechanisms comes from studies on small-molecule activators, which selectively modulate filter gating in C-type inactivating channels but are inactive in Kir channels (Schewe et al., 2019). Despite longer nonconductive phases in our simulations (Fig. S9), neither the ion occupancy in the SF nor the orientation of the carbonyl groups changed to “flipped” conformations (Fig. 7 F), as frequently observed in KcsA or K_v_ simulation studies (Holyoake et al., 2004; Cordero-Morales et al., 2006; Stansfeld et al., 2008), further supporting that the filter gating observed in GIRK2 is quite distinct from C-type inactivation. One important reason might lie in the different hydrogen-bond network behind the SF. Kir channels contain a highly conserved glutamate residue equivalent to KcsA residue E71 but lack the interacting aspartate or tryptophan residues. Instead, all Kir channels, except Kir7.1, possess a highly conserved arginine residue, which forms a salt bridge with the glutamic acid, as revealed by different Kir crystal structures (Fig. 7, D and E; Tao et al., 2009; Hansen et al., 2011; Whorton and MacKinnon, 2011, 2013; Lee et al., 2016).

In conclusion, our microsecond-scale simulations for the first time elucidate the elementary steps that underlie the movement of K^+^ ions through an inward-rectifier K^+^ channel under an applied electric field and provide a substantial extension to the conformational landscape available for GIRK channels.

## Supplementary Material

Supplemental Materials (PDF)

Video 1

Video 2

Video 3
