# Supplementary material for: Conduction through a narrow inward-rectifier K+ channel pore
Source: J Gen Physiol. 2019 Sep 11;151(10):1231–46. doi: 10.1085/jgp.201912359 (PMC6785732; doi:10.1085/jgp.201912359)
Supplement: Supplemental Materials (PDF) [file JGP_201912359_sm.pdf]

# Supplemental material

Bernsteiner et al., https://doi.org/10.1085/jgp.201912359

A

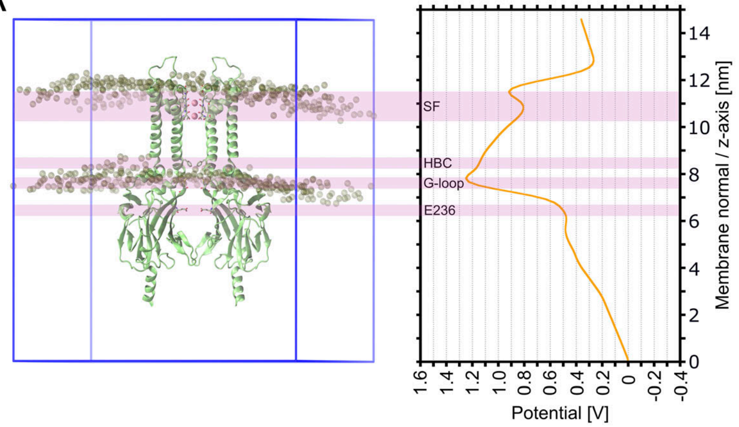


B

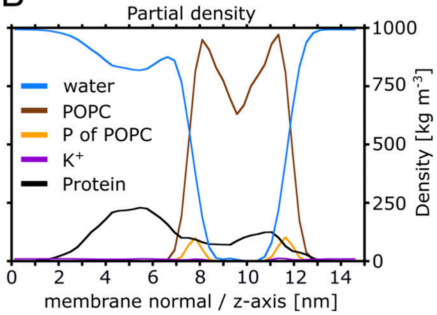


C

| | Ø Area per lipid [Å²] |
|---|---|
| MDs without field | 57.46 (± 1.80) |
| MDs with field | 57.15 (± 1.39) |

Figure S1. **Potential along the z axis and validation of the membrane integrity. (A)** Potential [V] along the z axis (membrane normal) of 1µs_run1 (applied electric field of 40 mV nm$^{-1}$ ~580 mV). The simulations box on the left side and the plot are aligned. Two subunits of the protein are shown. Phosphorus atoms of the membrane lipid POPC are visualized as brown spheres. **(B)** Partial density of the membrane, water, protein, and $K^+$ ions along the z axis (membrane normal) to verify membrane integrity (calculated for example MD: 1µs_run1). **(C)** Comparison of the average area per lipid (in Å$^2$) between MD runs with (14-µs data) or without (2-µs data) applied electric field.

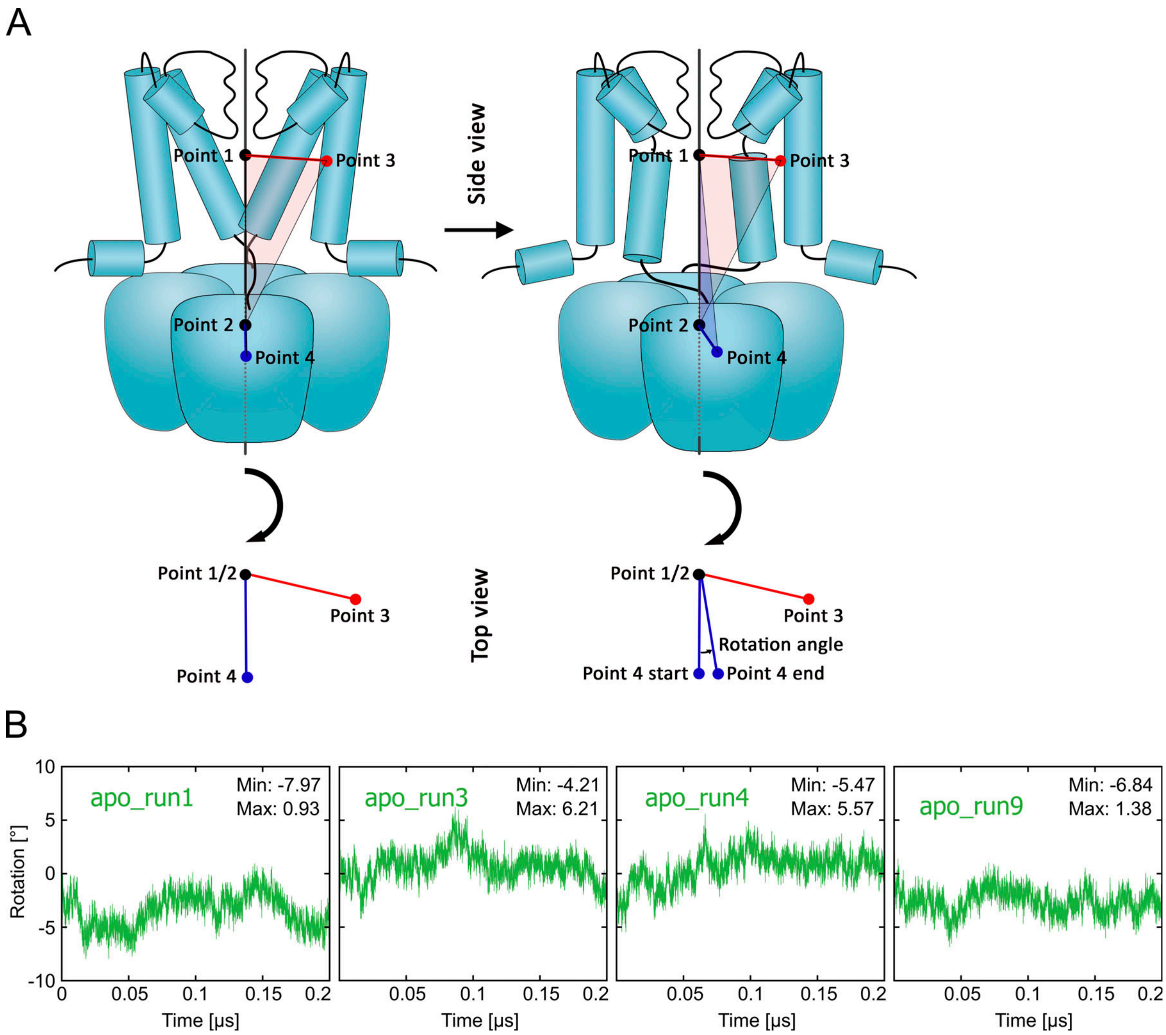


Figure S2. **Rotation of the CTD. (A)** Schematic figure, illustrating the points used to measure CTD rotation, as described in Materials and methods. **(B)** Angle of rotation of the CTD as a function of time for four $PIP_2$-depleted control runs that show a closing motion during 0.2 µs. The minimum and maximum angles during these 0.2-µs simulations are indicated.

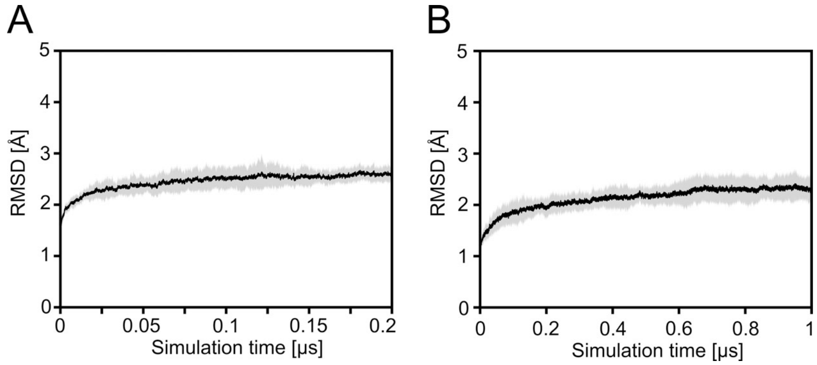


Figure S3. **RMSD of GIRK2 with bound $PIP_2$. (A)** Average RMSD of 10 × 0.2 µs. **(B)** RMSD of all unrestrained 1-µs runs (9-µs data). Both plots include SD (gray). The C-terminal helix (15 amino acids) was excluded from analysis.

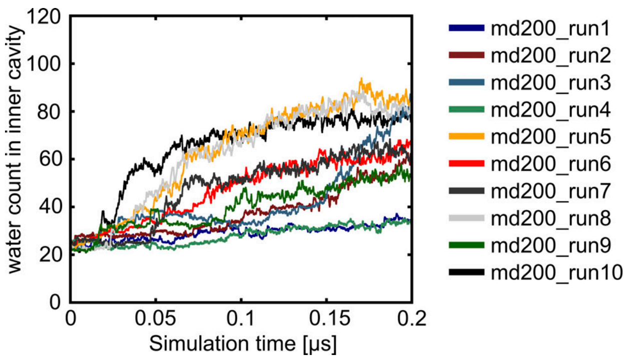


Figure S4. **Number of inner-pore water molecules as a function of simulation time.** The area between the four F192 side chains at the HBC gate and the selectivity filter was considered as inner pore. 10 × 0.2–µs runs are shown.

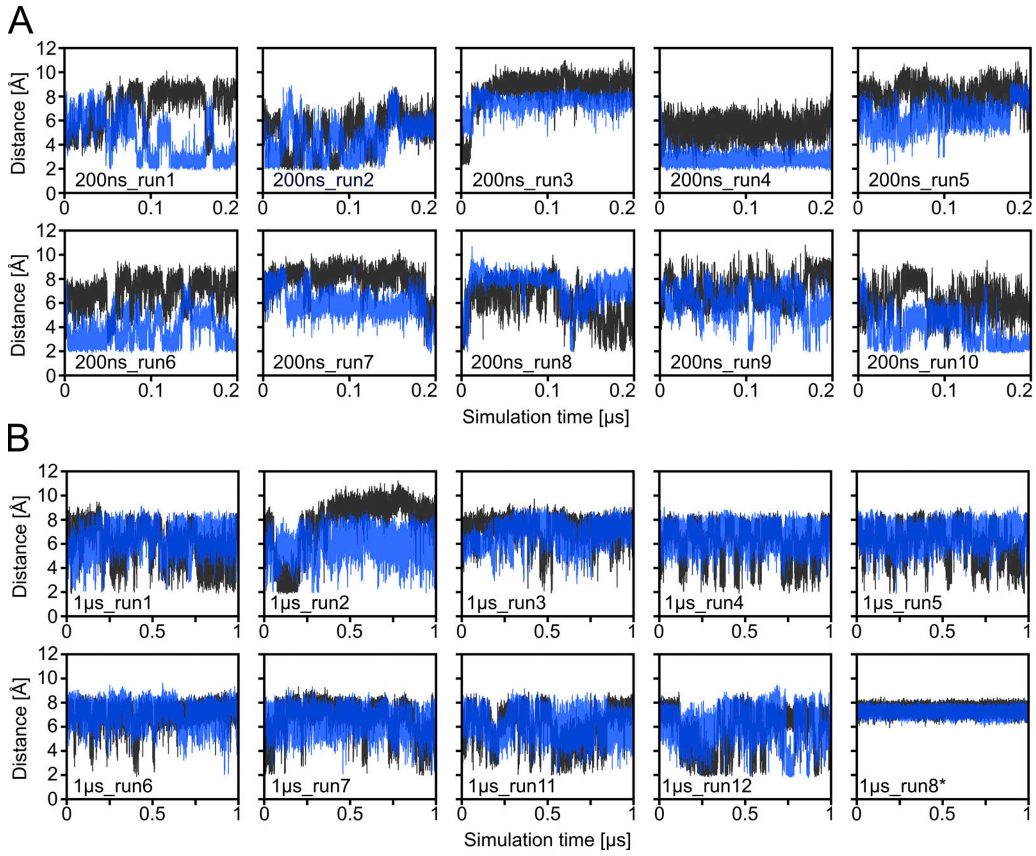


Figure S5. **Minimum distances at the G-loop gate.** Distances between opposing subunit pairs are plotted in black (pair1) and blue (pair2). **(A)** 10 × 0.2 µs. **(B)** 10 × 1 µs; the bottom right panel (1µs_run8[a]) shows the distance analysis of a restrained run (four more runs restrained in the same conformation are not shown).

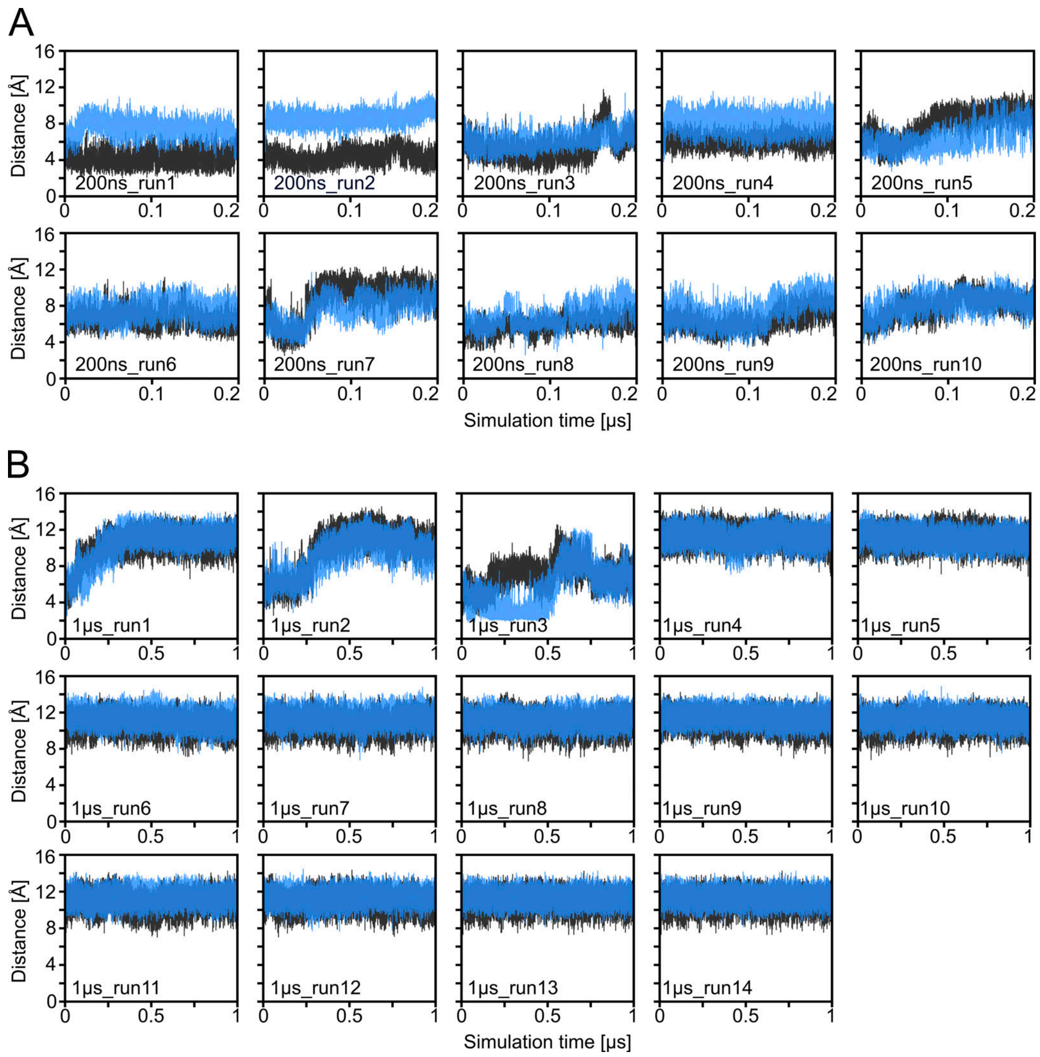


Figure S6. **Minimum distances at the HBC gate.** Distances between opposing subunit pairs are plotted in black (pair1) and blue (pair2). **(A)** 10 × 0.2 µs. **(B)** 14 × 1 µs.


https://doi.org/10.1085/jgp.201912359

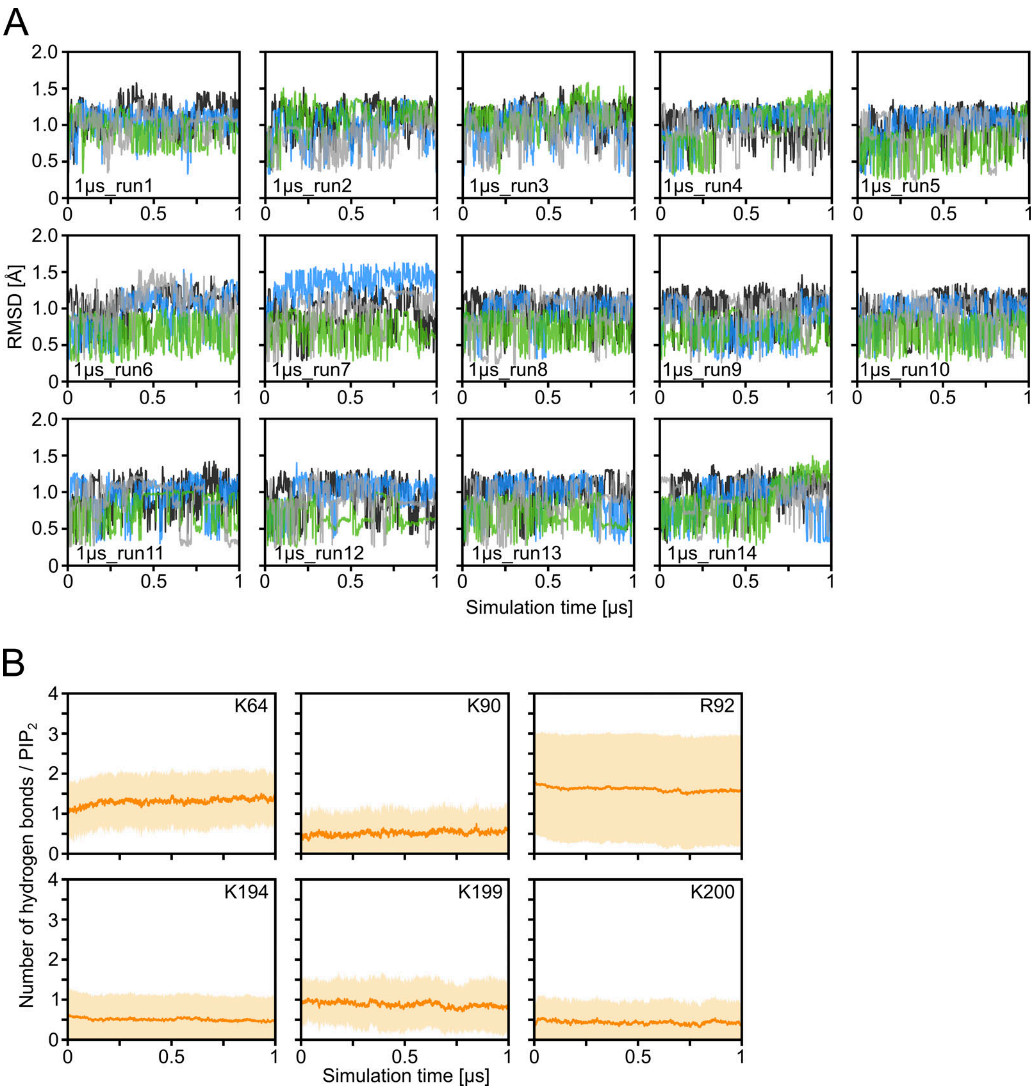


Figure S7. **Analysis of $PIP_2$ dynamics. (A)** RMSD analysis of $PIP_2$ for all 1-µs runs. Every color represents one of four $PIP_2$ molecules (black, gray, blue, and green). **(B)** Analysis of average number of hydrogen bonds with positively charged residues in the $PIP_2$ binding site, as shown previously by Whorton et al. (Whorton and MacKinnon, 2011). The results represent the combined simulation data of 14 × 1 µs with four subunits each and are shown as means ± SD.

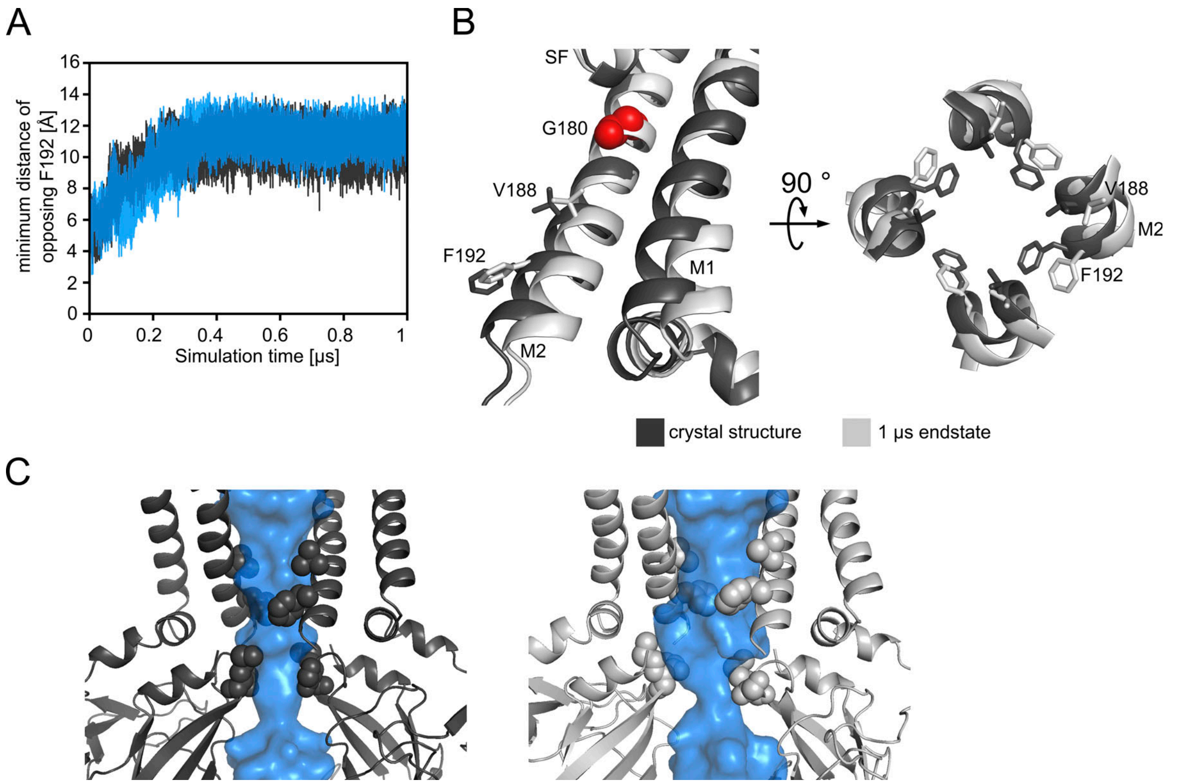


Figure S8. **Opening of the HBC gate caused by helix bending. (A)** Average minimum distance of opposing F192 residue pairs (black and blue plots), forming the narrowest part of the HBC gate, reveals an opening motion until a steady state is reached at ∼0.4 µs. The width of the black and blue plots (data for every 20 ps are included) indicate a certain amount of flexibility (>4 Å), even when steady state is reached. **(B)** Structural alignment of the crystal structure (PDB accession no. 3SYA, dark gray cartoon) with an example subunit of the end state (light gray cartoon) of the same 1-µs simulation presented in A, reveals a bending and slight twist of the transmembrane helices M1 and M2. The hydrophobic residues F192 and V188 face the inner pore in the crystal structure, while during MD, they move toward the neighboring channel subunit, leaving more space for solvation and ion permeation. Left: Side view. Right: Top view. **(C)** Channel interior surface (blue) calculated by the HOLLOW program (Ho and Gruswitz, 2008); left: crystal structure; right: 1-µs end state, showing an increase of the channel interior surface around the HBC gate.

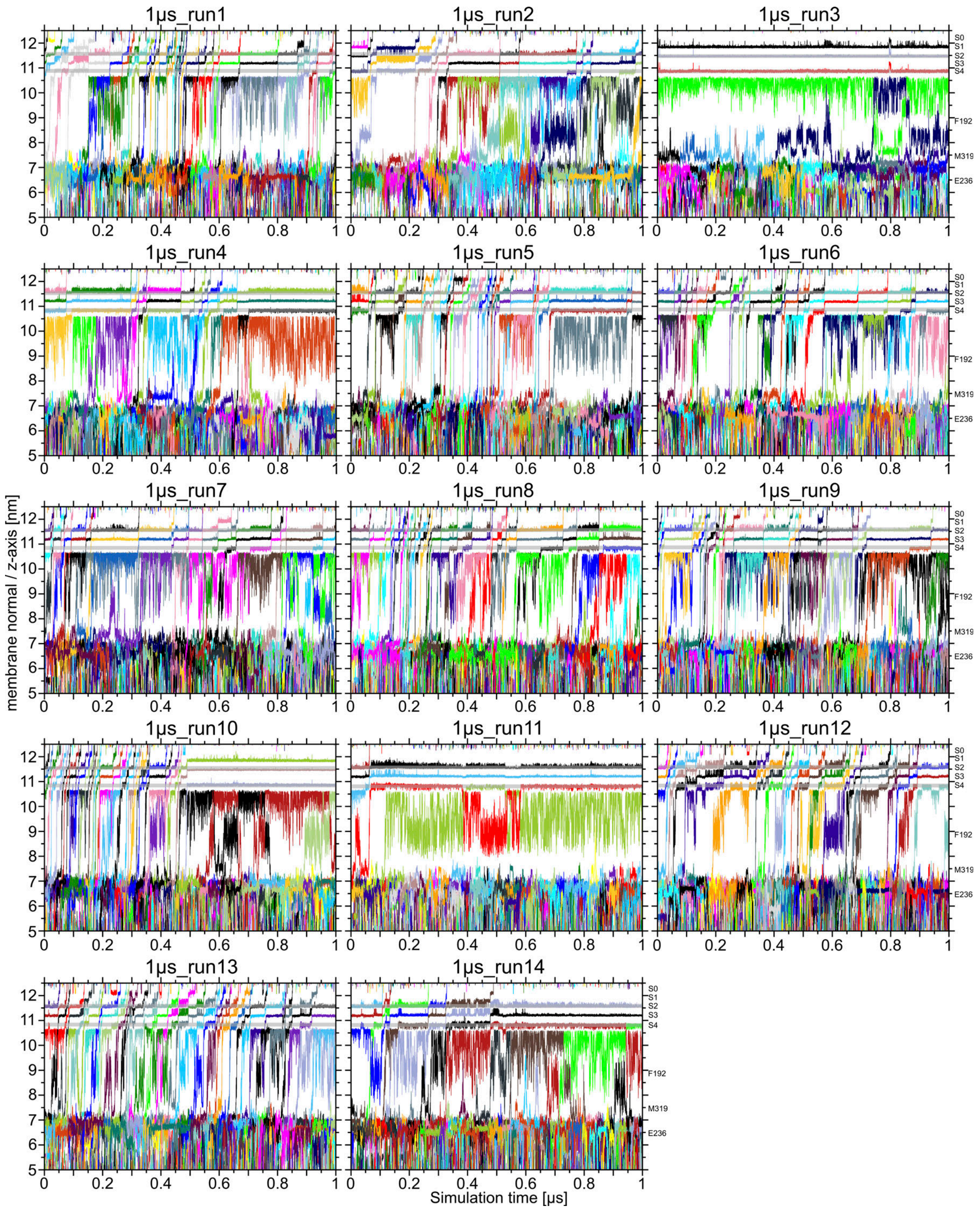


Figure S9. **Movement of ions through the channel.** Ions within the channel pore (along the membrane normal/z axis) as a function of simulation time for all 14 × 1-μs trajectories. The figure represents the channel cavity, starting at the intracellular entrance of the CTD until the end of the selectivity filter. Every colored line represents an individual ion.

JGP

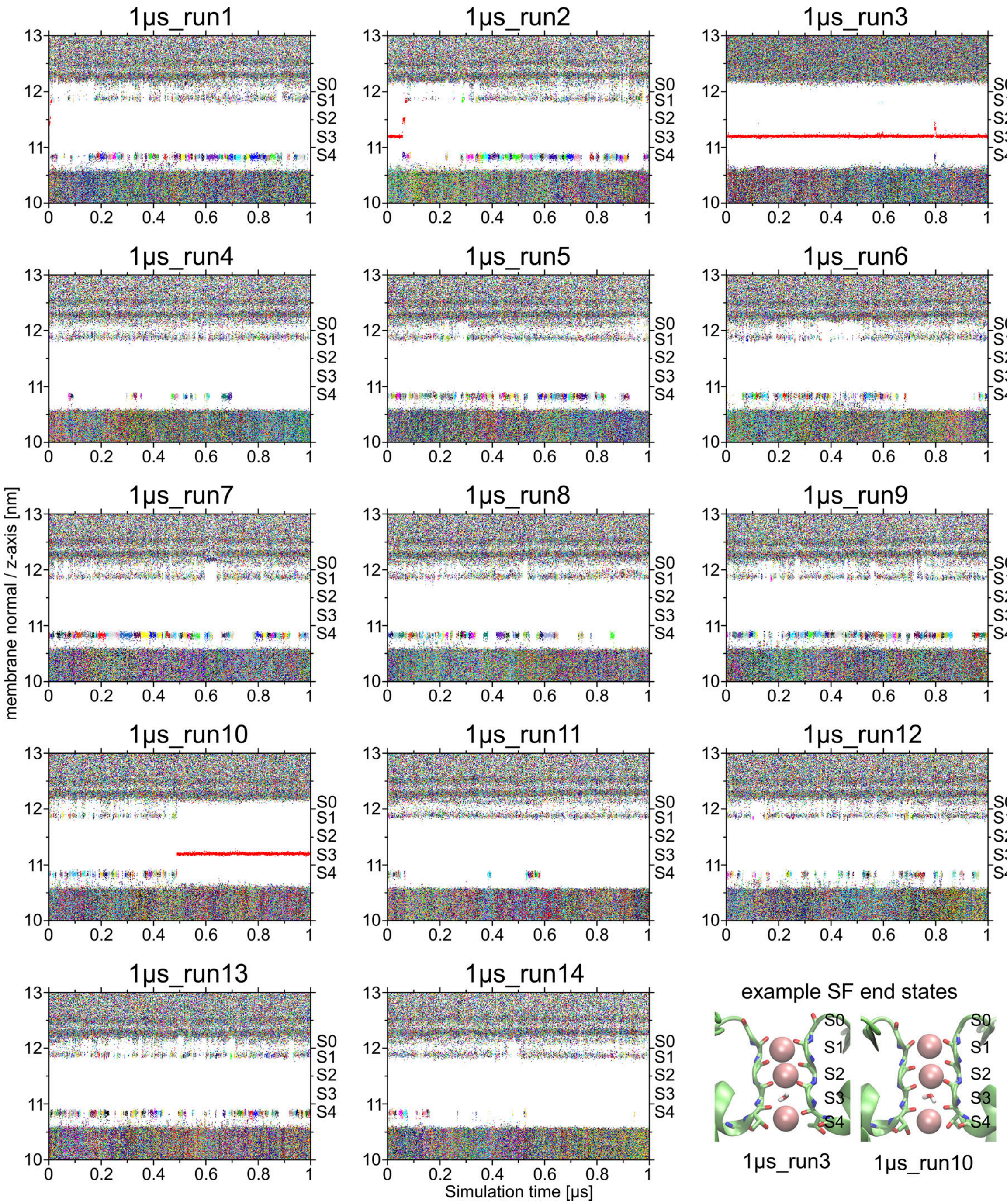


Figure S10. **Water in the selectivity filter.** Water along the membrane normal (z axis) as a function of simulation time for all 14 × 1-µs trajectories. Water molecules inside the selectivity filter, including small areas above and below, are included. The colored dots represent different water molecules. At the right side of each panel, positions S0–S4 are indicated. The bottom right corner shows two end states of the SF with a water at position S3 and ions at positions S1, S2, and S4. See also Fig. S9 for comparison.

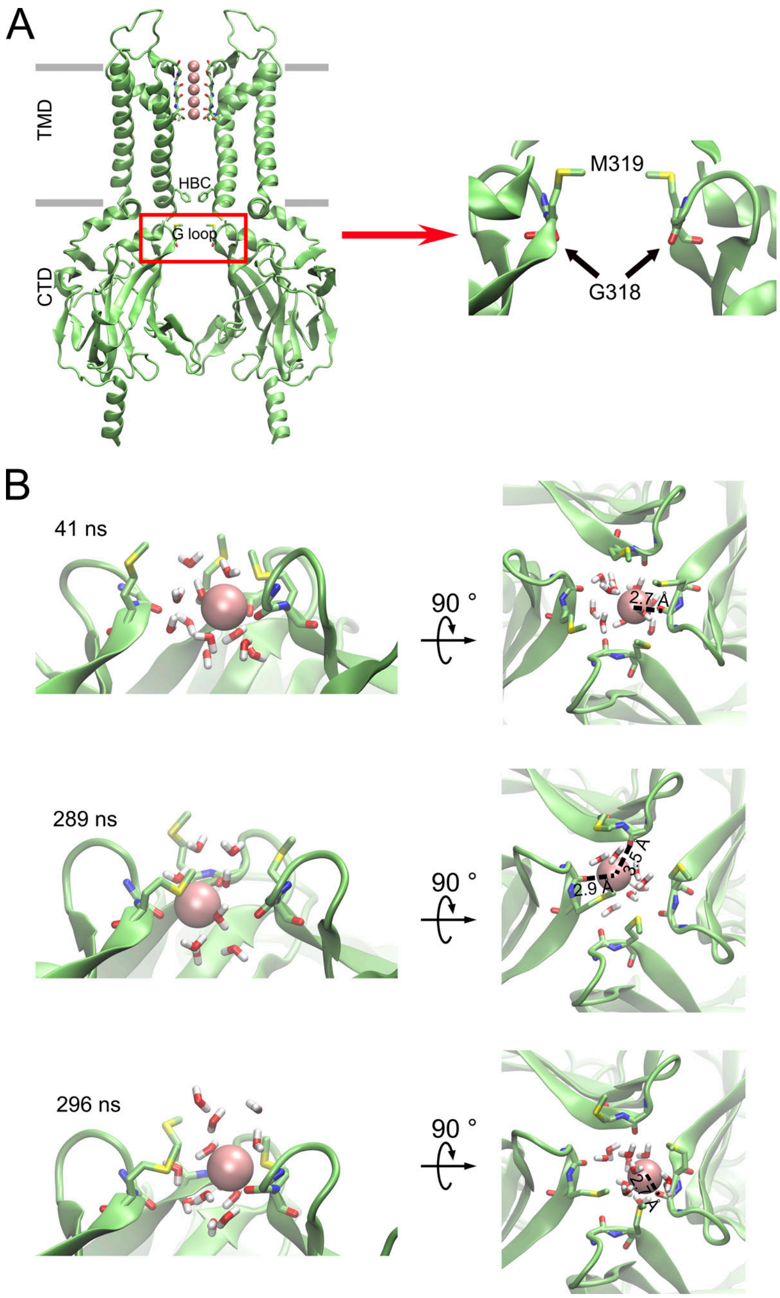


Figure S11. **Coordination of K⁺ ions by the carbonyl oxygen of G318. (A)** Overview of the GIRK2 structure. The G-loop gate is indicated by a red square. The gate-forming residues G318 and M319 are shown on the right side. **(B)** Three different example snapshots of how $K^+$ is coordinated by backbone oxygens of G318 at the relatively narrow G-loop gate. Left: Side views with front subunit hidden for better visibility. Right: Top views. The measured distances between $K^+$ and the close carbonyl oxygens of G318 are indicated. Water within 5 Å of the $K^+$ ion is shown.

Table S1. **Overview of control runs**

| MD run | Simulation time (μs) | Applied electric field (mV) |
|---|---|---|
| apo_run1 | 0.2 | 580 |
| apo_run2 | 0.2 | 580 |
| apo_run3 | 0.2 | 580 |
| apo_run4 | 0.2 | 580 |
| apo_run5 | 0.2 | 580 |
| apo_run6 | 0.2 | 580 |
| apo_run7 | 0.2 | 580 |
| apo_run8 | 0.2 | 580 |
| apo_run9 | 0.2 | 580 |
| apo_run10 | 0.2 | 580 |
| holo_run1 | 0.2 | 580 |
| holo_run2 | 0.2 | 580 |
| holo_run3 | 0.2 | 580 |
| holo_run4 | 0.2 | 580 |
| holo_run5 | 0.2 | 580 |
| holo_run6 | 0.2 | 580 |
| holo_run7 | 0.2 | 580 |
| holo_run8 | 0.2 | 580 |
| holo_run9 | 0.2 | 580 |
| holo_run10 | 0.2 | 580 |
| Total number | 4 | |

MD simulations with (holo) or without (apo) $PIP_2$ bound. Each run was performed with an applied electric field (40 mV $nm^{-1}$).

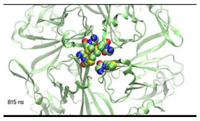


Video 1. **Permeation through a narrow G-loop gate.** Top view of the G-loop gate. Residues G318 and M319 are shown as spheres. The permeating $K^+$ ion is colored in dark purple. Water within 3.5 Å of $K^+$ is shown. The frame rate of the video is 25 frames per second.

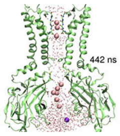


Video 2. **$K^+$ movement through the ion channel.** Side view of two subunits of Kir3.2. The permeating $K^+$ ion is colored in dark purple, and other $K^+$ ions are colored in pink. Water inside the channel pore is shown. The frame rate of the video is 25 frames per second.

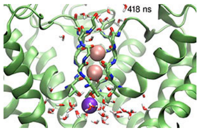


Video 3. **SF permeation via direct knock-on.** $K^+$ ions move through the SF in a fully desolvated manner. Permeating ions are alternatively colored in dark purple, pink, and ice blue. The frame rate of the video is 30 frames per second.

## References

Ho, B.K., and F. Gruswitz. 2008. HOLLOW: generating accurate representations of channel and interior surfaces in molecular structures. *BMC Struct. Biol.* 8:49. https://doi.org/10.1186/1472-6807-8-49

Whorton, M.R., and R. MacKinnon. 2011. Crystal structure of the mammalian GIRK2 K+ channel and gating regulation by G proteins, PIP2, and sodium. *Cell.* 147: 199–208. https://doi.org/10.1016/j.cell.2011.07.046
